# Neuron‐Targeted Exosomal Delivery of siRNA Against RIPK3 Slows Neurodegenerative Progression in Alzheimer's Disease

**DOI:** 10.1002/advs.76558

**Published:** 2026-07-11

**Authors:** Chi Zhang, Jiaqi Zhang, Yuzhi Wang, Yaodong Wang, Peng Lin, Yanling Wang, Zixuan Tang, Jintai Yu, Qin Zhou, Feiyun Cui

**Affiliations:** ^1^ School of Basic Medical Sciences Harbin Medical University Harbin Heilongjiang China; ^2^ The Heilongjiang Provincial Joint Laboratory of Basic Medicine and Multiple Organ System Diseases (International Cooperation) Harbin Heilongjiang China; ^3^ Heilongjiang Provincial Key Laboratory of Human Microphysiological Systems and Organoids‐on‐a‐Chip (Harbin Medical University) Harbin Heilongjiang China; ^4^ Department of Neurology and National Center For Neurological Disorders Huashan Hospital State Key Laboratory of Medical Neurobiology and MOE Frontiers Center For Brain Science Shanghai Medical College Fudan University Shanghai China

**Keywords:** blood–brain barrier (BBB), drug delivery, necroptosis, neuroprotection, RNA interference, synaptic plasticity

## Abstract

A major challenge in RNA therapeutics for central nervous system disorders is the lack of delivery systems capable of crossing the blood–brain barrier (BBB) while achieving cell‐type‐specific targeting. Herein, we develop an engineered exosomal siRNA delivery platform for systemic, neuron‐targeted RNA transport to the brain. The platform leverages exosomes derived from an immortalized mouse hippocampal neuronal cell line as a biomimetic and functionally privileged material source, enhancing neuronal uptake and intracellular delivery efficiency. Through surface functionalization with a rabies virus glycoprotein‐derived peptide, the system enables receptor‐mediated BBB transcytosis and programmable siRNA loading. In human cortical organoids, the platform achieves efficient cytosolic delivery and robust gene silencing in neurons, demonstrating high delivery precision and bioavailability. As a proof of concept, targeting receptor‐interacting protein kinase 3 (RIPK3) modulates necroptosis, a key pathway in inflammatory neurodegeneration. In transgenic mouse models, systemic administration suppresses RIPK3/MLKL signaling, reduces neuronal loss, and alleviates neuroinflammation and tau‐associated pathology. Transcriptomic analyses further indicate stabilization of neuronal homeostasis across vulnerable brain regions. Collectively, the study establishes a modular and programmable exosomal RNA delivery platform and highlights age‐defined, cell‐derived biomaterials as a generalizable strategy for overcoming delivery barriers in neurological diseases.

## Graphical abstract

Engineered exosomal siRNA delivery platform enables systemic, neuron‐targeted RNA transport across the blood–brain barrier. Surface functionalization with rabies virus glycoprotein‐derived peptide facilitates receptor‐mediated transcytosis, achieving efficient cytosolic delivery and robust gene silencing. Targeting RIPK3 suppresses necroptosis, reduces neuronal loss, and stabilizes neuronal homeostasis, establishing a modular strategy for overcoming delivery barriers in neurological diseases. Schematic illustration of the si‐*Ripk3*@Exo^RVG^‐mediated alleviation of cognitive impairment in Alzheimer's disease. Created in BioRender. Chi, Z. (2026) https://BioRender.com/x8sbwrb.

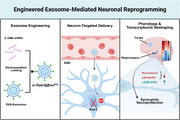

## 1 Introduction

Alzheimer's disease (AD) remains without an effective disease‐modifying therapy once it reaches moderate to severe stages. Although amyloid‐β (Aβ)–targeting immunotherapies have recently achieved regulatory approval and show measurable benefits in early disease, their clinical efficacy diminishes sharply as AD progresses, when extensive neuronal loss and structural brain atrophy are already established [1, 2]. This therapeutic limitation underscores a central challenge in AD treatment: once neurodegeneration is underway, strategies focused on upstream pathological aggregates are insufficient [3, 4]. Preserving neuronal survival and circuit integrity, therefore, represents a critical, yet largely unmet, therapeutic priority for advanced AD.

Accumulating evidence indicates that neuronal death in AD is not a passive consequence of degeneration but an actively regulated process [3]. Among multiple forms of programmed cell death, necroptosis has emerged as a particularly detrimental mechanism in neurodegenerative disease [5, 6]. Unlike apoptosis, necroptosis results in cell lysis and the release of damage‐associated molecular patterns (DAMPs), which activate microglia and perpetuate a vicious cycle of neuroinflammation [7, 8, 9]. Central to this pathway are receptor‐interacting protein kinase 1 and 3 (RIPK1/RIPK3), which execute necroptosis through phosphorylation of the terminal effector Mixed lineage kinase‐like protein(MLKL) [10, 11]. Elevated RIPK1/RIPK3 activity has been observed in AD brains and correlates with tau pathology and neuronal injury, positioning necroptosis as a mechanistic driver rather than a bystander of disease progression [3, 11, 12]. Therapeutically targeting necroptosis, however, presents a challenge. Upstream kinases such as RIPK1 have been explored as drug targets, yet their kinase‐independent scaffolding functions are essential for cell survival and inflammatory signaling, raising safety concerns [5, 13, 14]. In contrast, RIPK3 operates as a more specific execution node of necroptosis, offering the possibility of selectively blocking inflammatory cell death without disrupting essential homeostatic pathways. Despite this conceptual appeal, no effective strategy currently exists to achieve cell‐type‐specific RIPK3 inhibition within the central nervous system [15].

Small interfering RNA (siRNA) provides a highly specific and potent approach for gene silencing. Developing siRNA against *Ripk3* could effectively inhibit necroptosis. However, delivering therapeutic siRNA across the blood–brain barrier (BBB) remains a formidable obstacle [16, 17]. Conventional synthetic nanocarriers often fail to reconcile efficient BBB transport with neuronal targeting and long‐term biocompatibility, creating a persistent delivery bottleneck for central nervous system therapeutics. Current RNA delivery strategies are heavily constrained by these intrinsic hurdles: viral vectors suffer from immunogenicity and limited cargo capacity, while lipid nanoparticles are plagued by poor BBB penetration and prominent off‐target accumulation [18]. Endogenous exosomes offer a fundamentally different solution. As naturally occurring extracellular vesicles, exosomes exhibit low immunogenicity, intrinsic stability in biological fluids, and an inherent capacity to cross the BBB [19, 20, 21, 22]. Moreover, their biological origin enables efficient cellular uptake while minimizing off‐target toxicity. Engineering exosomes with targeting ligands further enhances their delivery precision. In particular, the rabies virus glycoprotein (RVG) peptide binds neuronal receptors and facilitates receptor‐mediated transcytosis across the BBB, enabling sequential targeting from brain endothelium to neurons [23, 24, 25, 26]. This strategy leverages endogenous trafficking pathways rather than forcing synthetic penetration, offering a potentially generalizable platform for brain‐targeted RNA delivery.

Herein, we report a neuron‐targeted exosomal RNA interference strategy to suppress necroptosis in AD. We engineered RVG‐functionalized exosomes loaded with siRNA against *Ripk3* (si‐*Ripk3*@Exo^RVG^) and evaluated their therapeutic efficacy across transgenic mouse models and human pluripotent stem cell‐derived cortical organoids. In the murine experiments, HT22 cells were selected as the exosome source to exclude the confounding anti‐inflammatory and reparative effects of MSC‐derived exosomes, thereby allowing a rigorous assessment of the system's therapeutic efficacy. Conversely, for validation in human cortical organoids, exosomes derived from HEK‐293T cells were utilized to circumvent the use of tumor‐derived human neuronal cell lines such as SH‐SY5Y, minimizing potential tumorigenic risks. We show that si‐*Ripk3*@Exo^RVG^ efficiently crosses the BBB, achieves cytosolic siRNA delivery, and selectively silences RIPK3 in neurons. This intervention attenuates necroptosis‐associated neuroinflammation, preserves synaptic architecture, and improves cognitive performance in advanced AD models. Transcriptomic analyses further reveal systems‐level reprogramming of neuronal signaling networks toward a pro‐survival state. Together, these findings identify necroptosis as a tractable therapeutic axis in late‐stage AD and establish engineered exosomes as a scalable nanotechnological framework for precision RNA delivery to the brain.

## 2 Results

### 2.1 Tau Pathology Drives Neuronal Injury Through RIPK3‐Dependent Necroptosis

**FIGURE 1:**
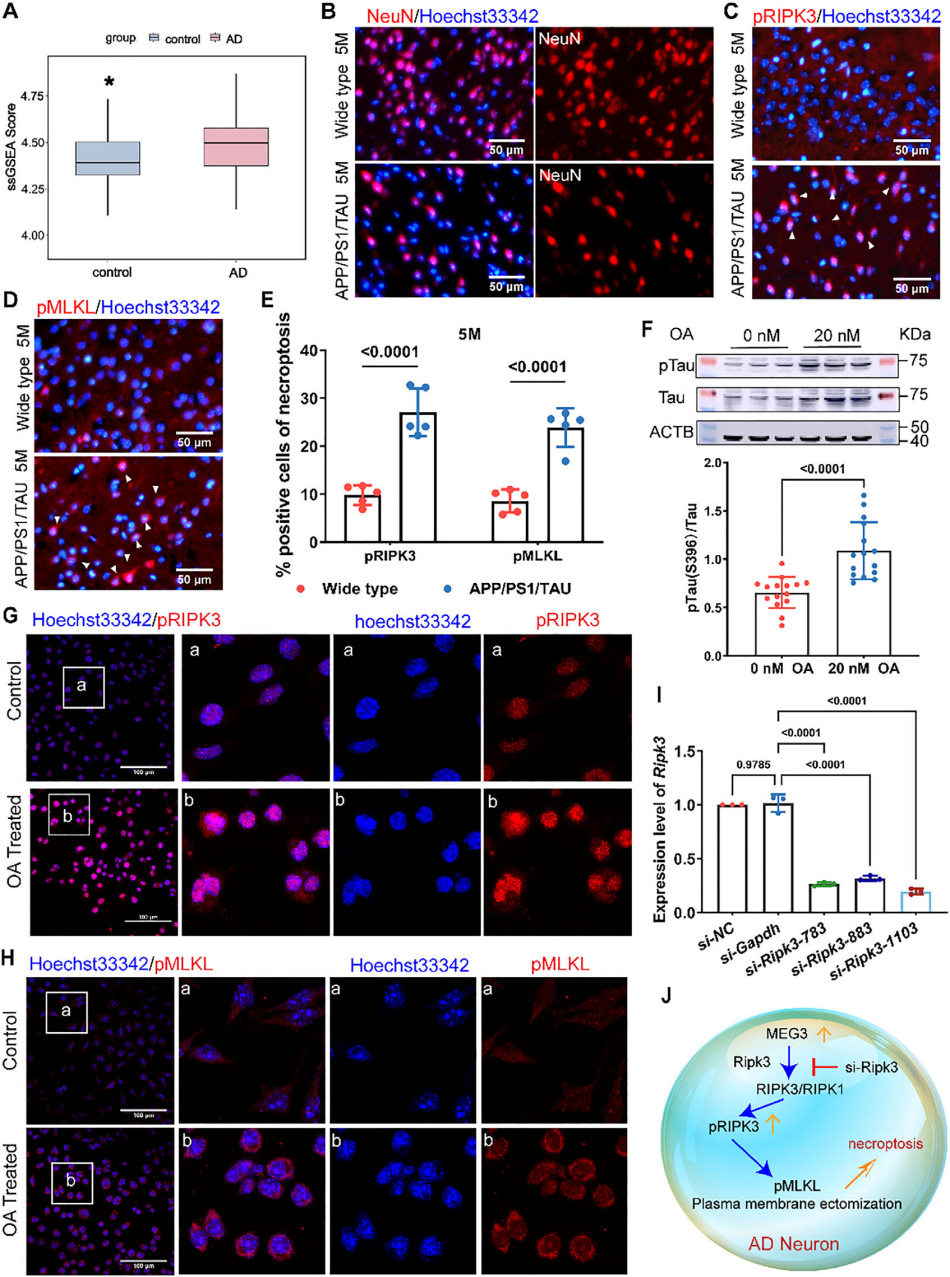
Bioinformatics analysis and in vitro experiments reveal the role of RIPK3‐mediated necroptosis in AD pathology. (A) ssGSEA analysis revealed that the necroptotic signaling pathway was enriched in the AD group. (B) Immunofluorescence staining revealed an apparent loss of NeuN‐positive neurons in the brain tissue of APP/PS1/TAU mice. Bar = 50 µm. (C) Immunofluorescence labeling revealed the distribution of pRipk3‐positive cells in the brain tissue of APP/PS1/TAU mice. (D) Immunofluorescence labeling revealed the distribution of pMLKL‐positive cells in the brain tissue of APP/PS1/TAU mice. Bar = 50 µm. In Figures C, D, arrows indicate the distribution of pRIPK3‐ or pMLKL‐positive cells. (E) The quantitative statistical positive cell percentage in Figures C, D, *n* = 5. (F) A representative western blotting image displaying the changes in protein expression (pTau, Tau, and ACTB) post‐OA treatment in HT22 cells, along with its corresponding quantitative analysis bar graph. Unpaired two‐tailed Student's *t*‐test, *n* = 5 independent experiments, each performed with 3 biological replicates. (G, H) Immunofluorescence staining of pRIPK3 (G) and pMLKL (H) expression in HT22 cells after OA treatment. Bar = 100 µm. (I) qPCR statistical graph depicting the effective silencing of *Ripk3* mRNA expression through siRNA sequences. Statistical analysis was performed using one‐way ANOVA followed by Tukey's post hoc test. (J) Schematic diagram illustrating RIPK3/MLKL‐mediated necroptotic signaling in AD neurons and the proposed inhibitory effect of si‐*Ripk3* on this pathway. In panel (E), data points represent brain sections from individual mice. In panels (F, I), data points represent independent replicates.

Neuronal loss in AD has traditionally been attributed to apoptotic mechanisms; however, increasing evidence indicates that necroptosis represents a distinct and highly relevant mode of inflammatory neuronal death in neurodegenerative conditions. Unlike apoptosis, which proceeds through an immunologically silent and orderly dismantling of cellular components, necroptosis is characterized by membrane rupture and the release of pro‐inflammatory signals, thereby amplifying tissue damage. This process is critically governed by the RIPK1‐RIPK3‐MLKL signaling axis. To explore the involvement of necroptosis in human AD pathology, we analyzed transcriptomic data from a frontal cortex cohort (GSE122063) comprising 136 postmortem samples using the GSVA package in R language. After quality control and stratification, 44 AD cases and 56 age‐matched control samples were included in the final analysis. Single‐sample gene set enrichment analysis (ssGSEA) revealed a significant upregulation of RIPK3/MLKL‐associated necroptotic gene signatures in AD patients compared with controls, indicating activation of necroptotic pathways in the diseased human brain (Figure 1A). These findings are consistent with previous reports demonstrating that aberrant necroptosis contributes to neuronal loss, cognitive decline, and brain atrophy in AD, and that excessive activation of necrotic signaling pathways is a major determinant of disease severity [5, 27].

We next examined whether necroptosis is engaged in vivo in a tau‐driven AD model. Histopathological analysis of FAD3T (APP/PS1/Tau) mice revealed pronounced neuronal loss in the cortex and hippocampus, accompanied by robust activation of RIPK3‐dependent necroptotic signaling (Figure 1B–E). Mechanistically, necroptosis requires RIPK3 activation through phosphorylation at conserved residues, followed by the formation of a death complex that promotes MLKL phosphorylation. Activated MLKL oligomerizes and translocates to the plasma membrane, where it disrupts membrane integrity and induces cell lysis. To further establish a mechanistic link between tau pathology and necroptosis, we employed an in vitro model in which oxalyl acid (OA) was used to induce tau hyperphosphorylation in HT22 cells. Conditions that increased the pTau/Tau ratio concomitantly triggered RIPK3 and MLKL phosphorylation, indicating that tauopathy is sufficient to activate necroptotic signaling in neurons (Figure 1F). The results indicated that OA doses capable of triggering an increase in pTau/Tau expression and also induced an increase in pRIPK3 and pMLKL expression in HT22 cells (Figure 1G,H). Collectively, our results support a model in which tau‐driven neurodegeneration in AD is mediated by RIPK3‐dependent neuron necroptosis. This mechanism provides a direct molecular link between tau pathology, inflammatory neuronal death and progressive neurodegeneration, highlighting necroptosis as a disease‐relevant and therapeutically tractable pathway in AD. Meanwhile, the multifunctional roles and knockout‐associated lethality of MLKL suggest that RIPK3 represents a more viable therapeutic target in advanced tauopathy‐associated AD, supporting si‐Ripk3 as a potential therapeutic strategy (Figure 1J).

### 2.2 Development and Characterization of RVG‐Enriched Hippocampal Neuron‐Derived Exosomes

Autologous exosomes are well suited as drug delivery vehicles owing to their intrinsic biocompatibility and efficient cellular internalization by recipient cells. In particular, neuron‐derived exosomes provide inherent advantages for central nervous system delivery due to their native tropism and reduced immunogenicity [28, 29]. To further enhance neuron‐specific targeting and facilitate BBB translocation, the RVG peptide was employed. RVG is known to bind nicotinic acetylcholine receptors expressed on neuronal cells and brain endothelial cells, enabling efficient brain targeting and neuronal uptake, and has therefore been widely used to functionalize nanocarriers for central nervous system–directed delivery.

To generate RVG‐modified exosomes, a lentiviral vector encoding the RVG‐Lamp2 fusion protein (pLV‐CMV‐RVG‐Lamp2‐3×Flag) was packaged in HEK293T cells by co‐transfection with pMD2.G and psPAX2. The resulting lentivirus was subsequently used to establish a stable HT22 cell line expressing RVG‐Lamp2 (designated HT22‐RVG), from which engineered exosomes (Exo^RVG^) were harvested (Figure S12A,B). Expression of the RVG‐Lamp2‐Flag fusion protein in HT22‐RVG cells was confirmed by western blot analysis of whole‐cell lysates (Figure S12C).

Next, Exos were isolated from HT22 and HT22‐RVG cells and then systematically characterized in terms of size distribution, morphology, and protein markers for Exo (TSG101 and CD63). Nanoparticle tracking analysis showed that both Exo and Exo^RVG^ exhibited a comparable size distribution, with mean diameters centered at approximately 140 nm (Figure S12D,E). Transmission electron microscopy with negative staining further confirmed that Exo^RVG^ displayed intact lipid bilayers and characteristic cup‐shaped or concave vesicular morphologies (Figure S12F,G). Further analyses confirmed that the isolated exosomes were positive for the canonical exosomal markers TSG101 and CD63, while lacking expression of the endoplasmic reticulum marker Calnexin (Figure S12H). Moreover, no significant differences were observed in surface zeta potential or electrophoretic mobility between Exo^RVG^ and unmodified Exo (Figure S12I,J), indicating that RVG engineering did not measurably alter the physicochemical properties of neuron‐derived exosomes.

### 2.3 Exo^RVG^ Efficiently Targets and Enters Neuronal Cells

To assess whether Exo^RVG^ possesses the prerequisite capability to traverse the BBB and subsequently engage neuronal targets, we adopted a two‐step in vitro evaluation strategy encompassing barrier penetration and cellular uptake. As an initial validation, an in vitro Transwell‐based BBB co‐culture model was established to recapitulate key structural and functional features of the neurovascular unit, incorporating bEnd.3 brain microvascular endothelial cells, MBVP pericytes, and astrocytes (Figure S1A). The integrity and functionality of the reconstructed barrier were confirmed by stable and high trans‐endothelial electrical resistance (TEER) values (Figure S1B).

Using this validated platform, permeability across the BBB model was first examined. A fluorescent tracer, serving as a proxy for PKH26‐labeled Exo^RVG^, was introduced into the apical (vascular) compartment. Quantitative analysis revealed that approximately 53.07% of the tracer signal was detected in the basolateral (brain parenchyma) compartment (Figure S1C,D). These results establish the suitability of the in vitro BBB model for evaluating exosome‐mediated delivery and support the feasibility of Exo^RVG^ transcytosis across the BBB.

To further determine cell‐type‐specific uptake, Exo^RVG^ was fluorescently labeled with PKH26 and incubated with recipient cells. Confocal fluorescence imaging revealed robust internalization of Exo^RVG^ by HT22 neuronal cells, whereas uptake by HEK293T cells was minimal (Figure S1E), consistent with RVG‐mediated neuronal targeting. These findings indicate that Exo^RVG^ exhibits preferential neuronal uptake, supporting its suitability as a neuron‐directed delivery vehicle. Finally, the biocompatibility of engineered exosomes was evaluated to exclude potential cytotoxic effects associated with vesicle isolation and modification. CCK‐8 assays demonstrated that exposure to Exo^RVG^ at concentrations up to 20 µg did not induce detectable cytotoxicity in recipient cells (Figure S1F), confirming the favorable safety profile of the delivery system for subsequent functional studies.

### 2.4 Exo^RVG^ Encapsulated si‐*Ripk3* Inhibits Neuronal Necroptosis In Vitro

**FIGURE 2:**
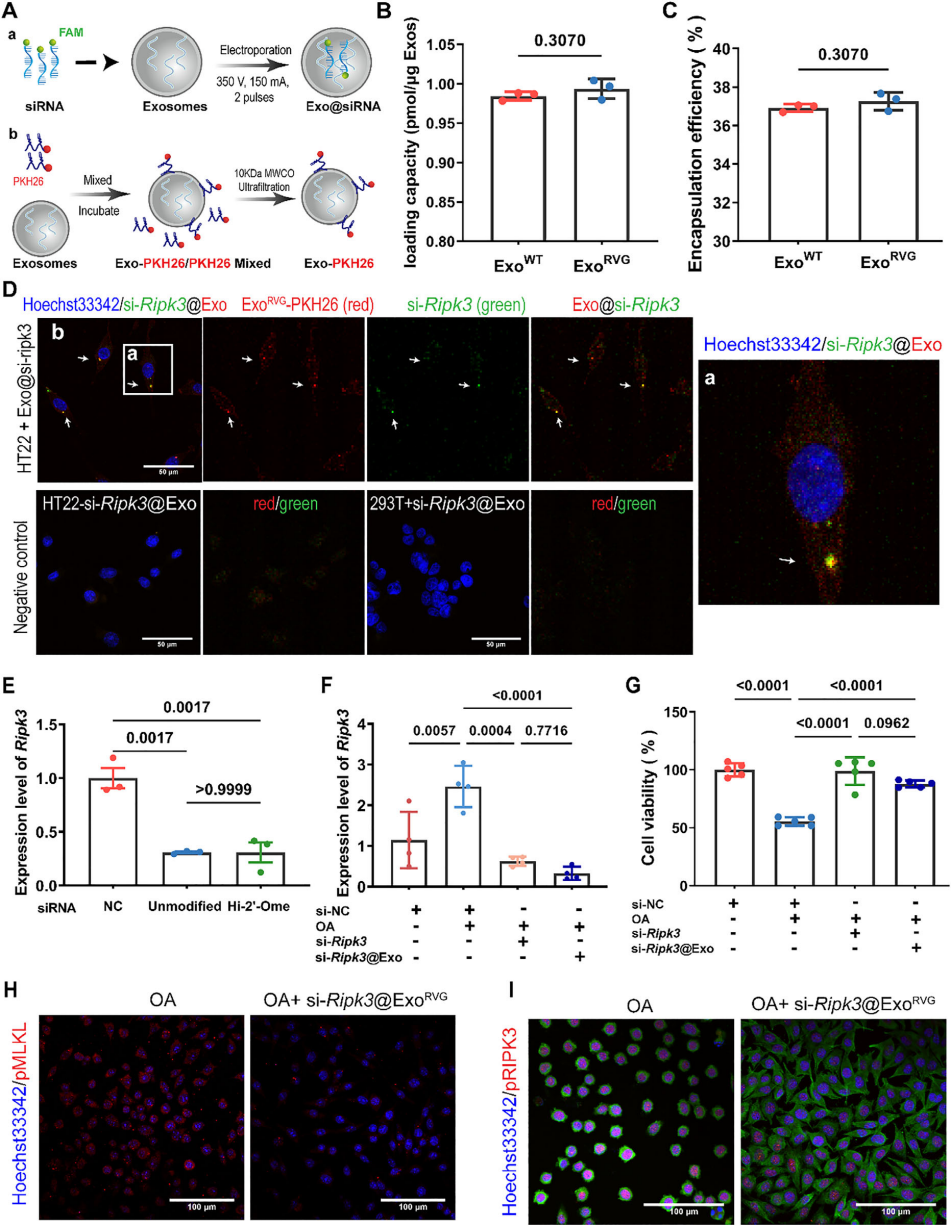
Exo^RVG^‐encapsulated si‐*Ripk3* suppresses necroptotic signalling in HT22 neurons. (A) Schematic illustration of siRNA loading into Exo^RVG^ by electroporation (a) and subsequent exosome labelling (b). (B) Quantification of siRNA loading capacity in Exo‐encapsulated formulation. (C) Quantification of encapsulation efficiency of Exo‐encapsulated siRNA. Both (B, C) were analyzed using unpaired t‐tests, and data points represent independent replicates. (D) SiRNA@Exo^RVG^ can be effectively internalized by HT22 cells. Bar = 50 µm. White arrows indicate positive signals for siRNA and Exo. (E) Validation of knockdown efficiency by qPCR for 2'‐OMe‐modified and unmodified si‐*Ripk3*. Unmodified: unmodified si‐Ripk3. Hi‐2'‐Ome: 2'‐O‐methyl‐modified si‐*Ripk3*. (F) Si‐*Ripk3*@Exo^RVG^ and si‐*Ripk3* effectively inhibit the upregulation of *Ripk3* in HT22 cells induced by OA. (G) Si‐*Ripk3*@Exo^RVG^ inhibits the effect of OA on the viability of HT22 cells. (H) Si‐*Ripk3*@Exo^RVG^ downregulated the OA‐induced pMLKL in HT22 cells. Bar = 100 µm. (I) Si‐*Ripk3*@Exo^RVG^ downregulated the OA‐induced pRIPK3 in HT22 cells. Bar = 100 µm. Statistical analysis was performed using a two‐tailed *t*‐test for (B, C) and one‐way ANOVA followed by Tukey's post hoc test for (E–G). Data points represent independent replicates (*n* ≥ 3).

Given the therapeutic potential of RIPK3 silencing for attenuating necroptosis in AD, the identification of an siRNA sequence with optimal silencing efficiency was a necessary prerequisite for functional validation. Herein, a panel of Ripk3‐targeting siRNAs was first screened in HT22 neuronal cells by transient transfection followed by quantitative PCR analysis. Among the candidates, si‐Ripk3‐883 achieved approximately 50% knockdown of Ripk3 mRNA and was therefore selected for subsequent studies (Figure 1I). Next, FAM‐labeled si‐Ripk3 was then loaded into Exo^RVG^ via electroporation, as schematically illustrated in Figure 2A. Loading efficiency and cargo content were quantified based on fluorescence measurements following ultrafiltration‐based separation of free and exosome‐associated siRNA. Both wild‐type exosomes and RVG‐engineered exosomes exhibited comparable siRNA loading capacities (∼0.98 pmol µg^−1^ exosomal protein) and encapsulation efficiencies ranging from 36% to 38% (Figure 2B,C), indicating that the engineering operation of RVG overexpression did not compromise siRNA loading. Confocal imaging further confirmed the successful co‐localization of Exo^RVG^ and si‐*Ripk3* within recipient cells (Figure 2D).

To enhance nuclease resistance and minimize TLR7/8‐mediated innate immune activation, si‐*Ripk3* was chemically modified with 2'‐O‐methyl (2'‐OMe) substitutions. This modification markedly improved siRNA stability while avoiding interference with Ago2 loading and RNA‐induced silencing complex (RISC) formation. Quantitative PCR analysis confirmed that 2′‐OMe–modified si‐*Ripk3* retained silencing efficacy comparable to the unmodified sequence (Figure 2E). Unless otherwise specified, subsequent experiments were performed using the modified si‐*Ripk3*.

The functional activity of si‐*Ripk3*@Exo^RVG^ was next evaluated in HT22 cells under OA‐induced necroptotic stress. Both si‐*Ripk3*@Exo^RVG^ and free si‐*Ripk3* effectively suppressed OA‐induced *Ripk3* upregulation at the mRNA level (Figure 2F), while preserving neuronal viability, as assessed by CCK‐8 assays (Figure 2G). Immunofluorescence analyses further revealed that si‐*Ripk3*@Exo^RVG^ markedly reduced phosphorylation of RIPK3 (Ser232) and MLKL (Ser358), key molecular events required for necroptosis execution (Figure 2H,I). Qualitative fluorescence intensity observations corroborated these reductions (Figures S2 and S3), solidifying effective blockade of necroptotic signaling. Collectively, these results demonstrate that Exo^RVG^‐mediated delivery enables efficient intracellular delivery and functional activity of si‐*Ripk3* in neurons, resulting in robust suppression of RIPK3‐dependent necroptosis in vitro. The enhanced efficacy of si‐*Ripk3*@Exo^RVG^ likely arises from stable siRNA encapsulation, protection from degradation and efficient cytosolic release, underscoring its suitability for subsequent in vivo applications.

### 2.5 Si‐*Ripk3*@Exo^RVG^ Rescues Neuronal Viability and Function Through Efficient Cytosolic Delivery and Coordinated Suppression of Necroptotic Signalling

**FIGURE 3:**
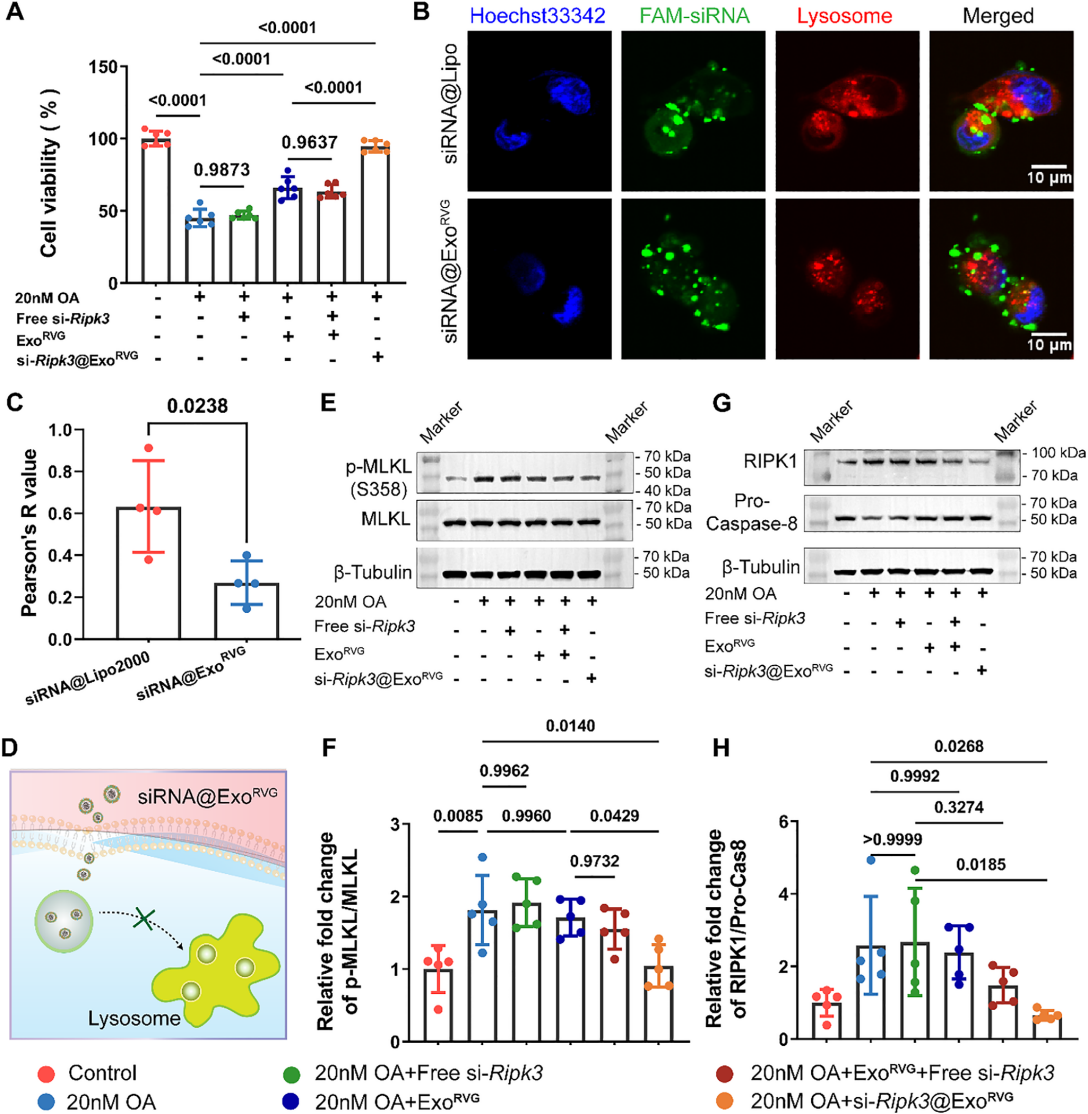
Si‐*Ripk3*@Exo^RVG^ rescues neuronal viability and function by silencing RIPK3 and facilitating cytosolic delivery of siRNA. (A) CCK‐8 assay was performed to evaluate the cell viability of HT22 cells treated with OA (oxaloacetic acid) to induce neurotoxicity. Treatment groups included control, OA alone, OA + free si‐*Ripk3*, OA + empty exosomes (Exo^RVG^), and OA + si‐*Ripk3*@Exo^RVG^. Only the si‐*Ripk3*@Exo^RVG^ group showed a complete restoration of cell viability to control levels. (B) Confocal microscopy images demonstrated the intracellular distribution of FAM‐labeled si‐Ripk3 (green) and lysosomes (Lysosome, red). Cell nuclei were counterstained with Hoechst 33342 (blue). The merged images show that in the siRNA@Exo^RVG^ group, the siRNA signal (green) is dispersed in the cytosol, separate from the lysosomal vesicles (red), confirming successful endosomal escape. (C) Quantitative analysis of the co‐localization between siRNA and lysosomes was performed using Pearson's correlation coefficient. The significantly lower R value in the siRNA@Exo^RVG^ group compared to the siRNA@Lipo2000 group indicates reduced lysosomal degradation and more efficient cytosolic delivery. Statistical analysis was performed using an unpaired *t*‐test, and data points represent results from independent representative images. (D) Schematic illustration of the endosomal escape mechanism of siRNA@Exo^RVG^. After cellular uptake, the siRNA cargo is released into the cytosol, avoiding degradation in lysosomes. (E) Western blot analysis revealed that si‐*Ripk3*@Exo^RVG^ significantly downregulated the expression of phosphorylated MLKL (pMLKL, phospho S358), a key mediator of necroptosis. (F) The bar graph shows the quantitative analysis results of the pMLKL/MLKL ratio. (G) Western blot analysis was used to assess the RIPK1/Pro‐Caspase‐8 ratio, another indicator of the necroptotic pathway. (H) The bar graph shows the quantitative analysis results of the RIPK1/Pro‐Caspase‐8 ratio. Legends for panels (F, G) are shown below. Data are presented as mean ± SD, with individual data points representing independent replicates (*n* ≥ 4). Statistical analysis was performed using one‐way ANOVA followed by Tukey's post hoc test.

Following confirmation of efficient si‐Ripk3 encapsulation within Exo^RVG^, we next evaluated its functional efficacy and underlying mechanism in an in vitro model of AD‐associated neuronal injury. Exposure of HT22 cells to OA, which induces tau hyperphosphorylation and necroptotic stress, resulted in a marked reduction in neuronal viability (Figure 3A). While treatment with empty exosomes conferred modest protection, it failed to fully counteract OA‐induced cytotoxicity. In contrast, treatment with pre‐encapsulated si‐*Ripk3*@Exo^RVG^ restored neuronal viability to levels comparable to untreated controls, indicating a substantially enhanced protective effect.

This improved efficacy was attributable to a dual and coordinated mechanism. First, si‐*Ripk3*@Exo^RVG^ achieved effective target engagement, as reflected by a pronounced reduction in the phosphorylation of MLKL at Ser358, a key executioner of necroptosis (Figure 3E,F). Given the established role of caspase‐8 as a negative regulator of necroptosis through cleavage of RIPK1 and RIPK3 [14], a decreased RIPK1/pro‐caspase‐8 ratio further indicated a shift in the intracellular environment away from a pro‐necroptotic state (Figure 3G,H) [30]. Crucially, confocal microscopy provided direct visual evidence for efficient cytosolic delivery of the siRNA cargo. Co‐localization analysis demonstrated a significant reduction in the overlap between FAM‐labelled si‐Ripk3 and lysosomal compartments following Exo^RVG^‐mediated delivery (Figure 3B–D), indicating successful escape from endo‐lysosomal sequestration. This step is essential for the functional incorporation of siRNA into the RNAinduced silencing complex and effective gene silencing.

To delineate the respective contributions of the exosome carrier and the siRNA cargo, a critical control was established in which free si‐*Ripk3* was physically mixed with empty exosomes (Exo^RVG^ + free si‐*Ripk3*). The CCK‐8 assay revealed that, although this mixture conferred a modest protective effect, the pre‐encapsulated si‐*Ripk3*@Exo^RVG^ complex exhibited significantly superior efficacy (Figure 3A). Notably, empty Exo^RVG^ also exerted a mild protective effect, likely reflecting the intrinsic trophic and cytoprotective cargo of neuron‐derived exosomes, which partially alleviates cellular stress but is insufficient to fully inhibit necroptosis [31, 32]. These findings indicate that the therapeutic advantage is not attributable merely to the presence of exosomes per se, but rather depends on the efficient intracellular delivery of si‐*Ripk3* enabled by exosomal encapsulation.

Notably, the neuroprotective effect of si‐*Ripk3*@Exo^RVG^ exceeded the additive effects of empty exosomes and free si‐Ripk3 alone (Figure 3A), indicating a synergistic interaction between the delivery vehicle and its RNA cargo. Together, these findings demonstrate that Exo^RVG^‐mediated si‐Ripk3 delivery integrates efficient cytosolic release with potent suppression of necroptotic signalling, thereby restoring neuronal viability and functional integrity under tauopathy‐associated stress.

Despite the promising in vitro results, the model cannot fully replicate the complex dynamic environment found in vivo. To obtain direct and visual preclinical evidence supporting the ability of Exo^RVG^ to traverse the BBB and accumulate within the brain parenchyma, we performed small‐animal in vivo imaging experiments (Figure S13A). Following the tail‐vein administration of DiR‐labeled Exo^RVG^, brain accumulation was readily visualized using an in vivo imaging system (Figure S13B). Quantitative analysis revealed that brain accumulation peaked approximately 6 h post‐injection, and the signal intensity in the brain was significantly higher for exosome‐encapsulated siRNA compared to free siRNA (Figure S13C). At 24 h post‐injection, DiR fluorescence was predominantly detected in the liver, while the accumulation rate of free siRNA in the spleen was markedly higher than that of exosome‐loaded siRNA (Figure S13D). These biodistribution profiles indicate that siRNA@Exo^RVG^ efficiently penetrates the BBB and accumulates within the brain parenchyma, while also exhibiting a more favorable systemic distribution than free siRNA. Importantly, Exo^RVG^ demonstrated superior brain‐targeting efficiency and prolonged cerebral retention, with maximal brain enrichment occurring within approximately 6 h after administration. Notably, the total radiant efficiency of the brain enrichment signal was approximately 2 × 10^9^ (p/s) / (µW/cm^2^) at around 6 h (Figure S13C), whereas imaging results at 24 h showed a total radiant efficiency of less than 1 × 10^9^ (p/s) / (µW/cm^2^) (Figure S13D). This ∼50% reduction suggests effective systemic clearance over time, supporting a favorable metabolic profile and preliminary in vivo safety of the Exo^RVG^‐based delivery system.

### 2.6 Si‐*Ripk3*@Exo^RVG^ Delays the Progression of Cognitive and Memory Decline in FAD3T Mice

To evaluate the therapeutic effects of si‐*Ripk3*@Exo^RVG^ in AD pathophysiological models in vivo, we performed a comprehensive behavioral assessment in FAD3T transgenic mice (carrying humanized *APP*, *PSEN1*, and *MAPT* genes with Swedish mutation, M146V mutation, and P301L mutation, respectively). This model recapitulates key pathological features of AD, including accelerated amyloid‐β deposition, severe tau hyperphosphorylation, synaptic loss and robust cognitive impairment, surpassing the pathological severity observed in single‐gene transgenic or non‐genetic AD mouse models. Notably, extensive cortical and hippocampal tau pathology and amyloid burden emerge as early as 2 months of age, and approximately 25% mortality is observed by 4 months. Given this aggressive and early‐onset disease course, 3‐month‐old FAD3T mice were selected for therapeutic intervention.

**FIGURE 4:**
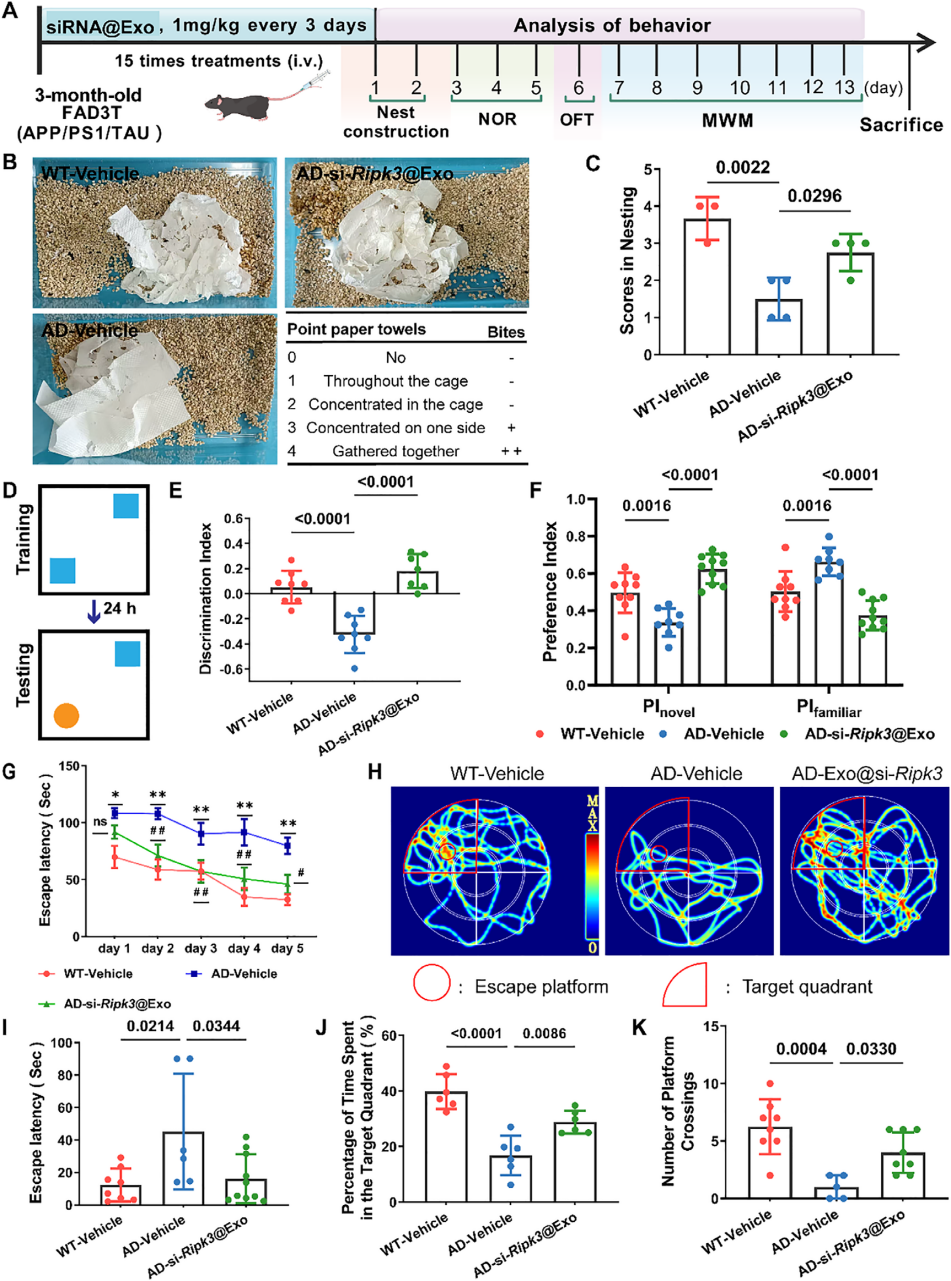
Si‐*Ripk3*@Exo^RVG^ delayed the progression of cognitive and memory decline in FAD3T^APP/PS1/TAU^ mice. (A) Schematic diagram of the process for siRNA@Exo treatment and behavioral analysis. (B) Representative images and scoring criteria of the nesting experiment for FAD3T mice and the control wild‐type (WT) mice. The photos were taken 24 h after nesting materials were introduced into the mice's housing cages. (C) Nest‐building scores for each group. (D) Setup for Novel Object Recognition Test. (E, F) Results for NOR test. DI (E) and PI (F) of each group after treatment. (G–K) Data for the probe test in the MWM. (G) The latency duration during training varied among the different treatment groups, reflecting their performance during days 1–5. Compared with the WT group, *p* < 0.05, ^**^ compared with the WT group, *p* < 0.01; # Compared with the AD‐ vehicle group, *p* < 0.05, ## compared with the AD‐vehicle group, *p* < 0.01; ns, no significant difference. (H) Trajectory maps of each group in the water maze, with red circles marking the location of the escape platform and red sectors indicating the target quadrants. (I) Statistical analysis of escape latency in each group during the testing phase. (J) The percentage of time spent in each target quadrant reveals significant differences between groups. (K) The analysis of platform crossing counts among various treatment groups indicated significant differences among them. Statistical analysis was performed using one‐way ANOVA followed by Tukey's post hoc test. In panel (C), data points represent independent cages; for all other statistical results, data points represent individual mice.

To determine whether si‐*Ripk3*@Exo^RVG^ could ameliorate AD‐associated neurobehavioral deficits, FAD3T mice received intravenous injections of si‐*Ripk3*@Exo^RVG^ via the tail vein at an siRNA dose of 1 mg/kg (exosomal protein) every 3 days (Figure 4A). Age‐matched vehicle‐treated FAD3T mice and wild‐type (WT) littermates were included as disease and baseline controls, respectively. Behavioral assessments encompassed nest‐building, open field, novel object recognition (NOR), and Morris water maze (MWM) tests to comprehensively evaluate hippocampus‐dependent functions, including motivation, non‐spatial memory, and spatial learning and memory [33, 34]. Nest‐building behavior, a sensitive indicator of hippocampal integrity and general well‐being, was profoundly impaired in vehicle‐treated FAD3T mice. In contrast, si‐*Ripk3*@Exo^RVG^ treated mice exhibited nest‐building scores comparable to those of WT controls and markedly higher than FAD3T controls (Figure 4B,C). Consistently, open field testing revealed a significant restoration of rearing (standing) frequency in si‐*Ripk3*@Exo^RVG^ treated mice relative to vehicle‐treated FAD3T mice (Figure S4B–D). Rearing behavior requires the integration of vestibular, somatosensory, and spatial information and is critically dependent on intact hippocampal‐prefrontal circuitry. These findings indicate that si‐*Ripk3*@Exo^RVG^ treatment preserves functional integrity within hippocampal neural networks in FAD3T mice. In the NOR test, vehicle‐treated FAD3T mice displayed a markedly reduced preference for novel objects compared with WT mice, as reflected by significantly lower discrimination index (DI) and preference index (PI) (Figure 4D–F). DI and PI are sensitive early indicators of non‐spatial recognition memory deficits and reflect dysfunction within the entorhinal cortex‐hippocampal circuit. Notably, si‐*Ripk3*@Exo^RVG^ treatment substantially improved NOR performance, with DI and PI values approaching those observed in WT mice (Figure 4E,F). These results suggest a robust rescue of hippocampus‐dependent non‐spatial memory, likely associated with improved synaptic plasticity and circuit‐level repair following necroptosis inhibition. Spatial learning and memory were further evaluated using the Morris water maze. During the 5‐day acquisition phase, all experimental groups exhibited comparable escape latencies, indicating intact learning ability (Figure 4G). In the probe test, vehicle‐treated FAD3T mice demonstrated disorganized search patterns and spent significantly less time in the target quadrant, reflecting impaired spatial memory retention (Figure 4H–J). In contrast, FAD3T mice receiving si‐*Ripk3*@Exo^RVG^ treatment exhibited a marked increase in target quadrant occupancy and a higher number of platform crossings relative to vehicle‐treated controls (Figure 4J,K). Moreover, the latency to first entry into the former platform location was significantly reduced in si‐*Ripk3*@Exo^RVG^ treated mice (Figure 4I), indicating enhanced memory retrieval. Collectively, these behavioral outcomes demonstrate that si‐*Ripk3*@Exo^RVG^ enables efficient brain delivery of therapeutic siRNA, significantly delays cognitive decline, and restores hippocampus‐dependent learning and memory in FAD3T mice. These findings provide compelling in vivo evidence supporting necroptosis as a viable therapeutic target in AD and highlight the translational potential of siRNA@Exo^RVG^ nanomedicine.

### 2.7 The Assessment of Cytotoxicity and In Vivo Biocompatibility of siRNA@Exo^RVG^

**FIGURE 5:**
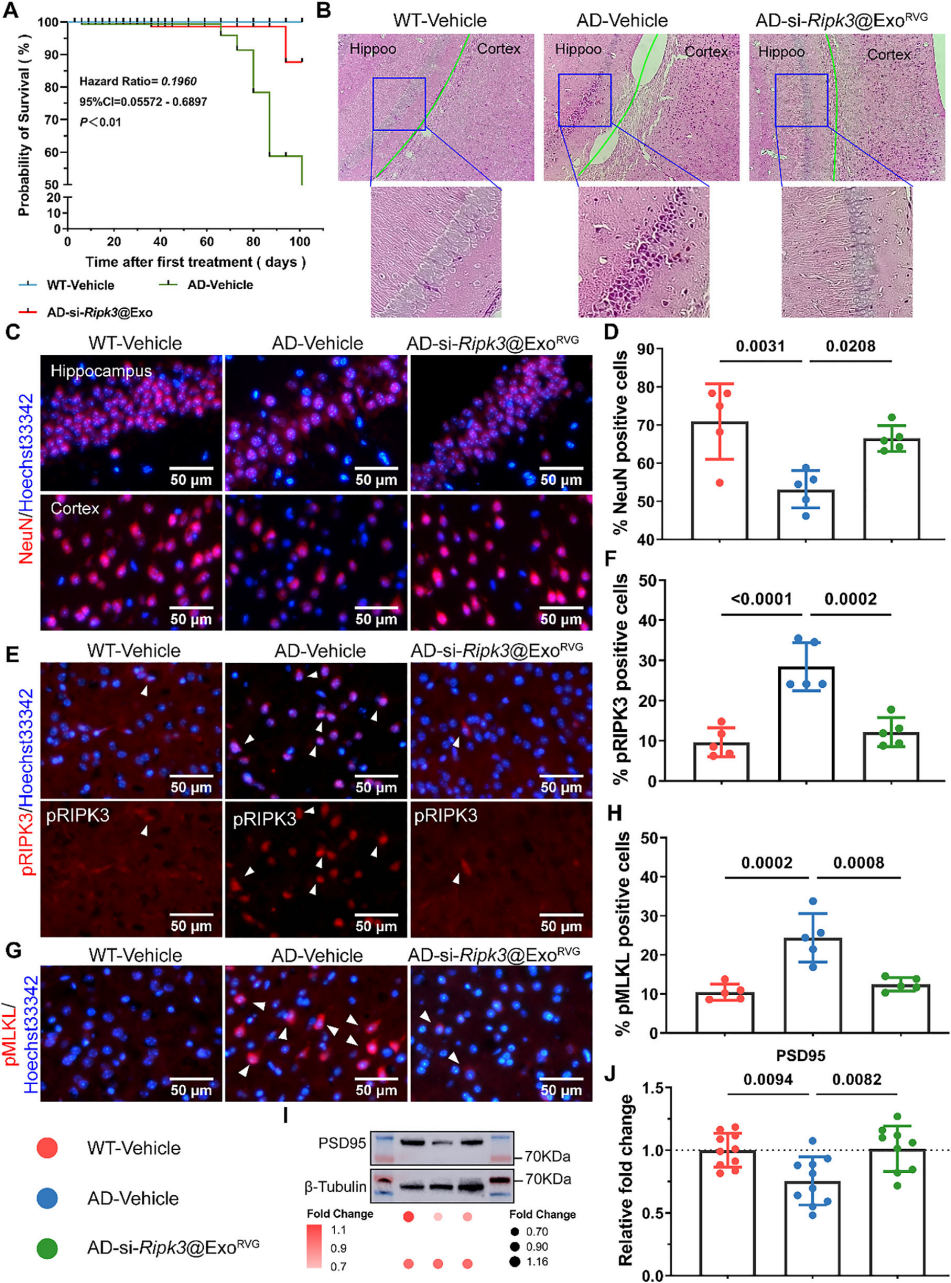
Si‐*Ripk3*@Exo^RVG^ protects neurons by inhibiting necroptosis and exhibits good biocompatibility in AD mice. (A) Survival curve illustrating the survival probabilities at various time points following the initial treatment for mice in three distinct treatment groups. The green line indicates the boundary between the cortex and the hippocampus. (B) Representative data of hematoxylin and eosin (HE) staining of the brain from APP/PS1/TAU mice treated with si‐*Ripk3*@Exo^RVG^ or vehicle and control wild‐type (WT) mice in 15 injection treatment experiments. (C) Immunofluorescent analysis of (4% PFA) fixed frozen OCT‐embedded mouse brain tissue using Anti‐NeuN antibody at a dilution of 1:200 and Cy3‐Conjugated AffiniPure Goat Anti‐Rabbit IgG(H+L). (D) The percentage of NeuN‐positive cells within the region of interest (ROI) (as depicted in Figure C). (E) Immunofluorescent analysis of (4% PFA) fixed frozen OCT‐embedded mouse brain tissue using Anti‐RIPK3 (phospho S232) antibody at a dilution of 1:200 and Cy3‐Conjugated AffiniPure Goat Anti‐Mouse IgG(H+L). White triangles indicate positive signals; the diffuse background represents nonspecific staining under low‐signal conditions. (F) The percentage of pRIPK3‐positive cells within the ROI (as depicted in Figure E). (G) Immunofluorescent analysis of (4% PFA) fixed frozen OCT‐embedded mouse brain tissue using Anti‐MLKL (phospho S345) antibody at a dilution of 1:1000 and Cy3‐Conjugated AffiniPure Goat Anti‐Rabbit IgG(H+L). (H) The percentage of pMLKL‐positive cells within the ROI (as depicted in Figure G). Bar = 50 µm. (I) Western blot analysis of the synaptic marker PSD95. (J) Quantification reveals increased PSD95 expression in si‐*Ripk3*@Exo^RVG^‐treated mice. All statistics were analyzed using one‐way ANOVA followed by Tukey's post hoc test, with *n* ≥ 5. In panels (D, F), data points represent brain sections from individual mice. In panel (J), data points represent independent replicates.

To comprehensively evaluate the long‐term safety profile of si‐*Ripk3*@Exo^RVG^, survival analysis was first performed. Kaplan–Meier analysis revealed that mice treated with si‐*Ripk3*@Exo^RVG^ exhibited a significantly reduced risk of mortality compared with the AD model group (hazard ratio [HR] = 0.1960, 95% confidence interval [CI] = 0.05572‐0.6897, *p* < 0.01), indicating a pronounced survival benefit (Figure 5A). Importantly, no significant difference in overall survival was observed between the si‐*Ripk3*@Exo^RVG^ treated group and wild‐type controls, suggesting that the treatment does not confer additional mortality risk and exhibits excellent systemic safety.

To further evaluate the in vivo biocompatibility and potential systemic toxicity, a comprehensive panel of plasma biochemical markers was examined, including alanine aminotransferase (ALT) and aspartate aminotransferase (AST) for hepatic function, as well as blood urea nitrogen (BUN), uric acid (UA), and creatinine (CR) for renal function. Biochemical profiling revealed no significant alterations in any of these parameters following si‐*Ripk3*@Exo^RVG^ administration (Figure S14B,C), indicating the absence of liver or kidney toxicity. In parallel, to evaluate treatment‐associated inflammatory responses, pro‐inflammatory cytokines (IL‐1β, IL‐6, and TNF‐α) were quantified in liver and kidney tissues. No significant elevations were observed compared with control groups (Figure S14D,E), effectively excluding treatment‐induced systemic or organ‐specific inflammation. Consistent with these findings, histopathological examination of major organs, including the heart, liver, spleen, lungs, and kidneys, revealed preserved tissue architecture without evidence of necrosis, fibrosis, or inflammatory infiltration (Figure S14A). Notably, hematoxylin and eosin (H&E) staining of brain sections further confirmed the absence of overt inflammatory responses in both the cortex and hippocampus following treatment (Figure 5B). These findings indicate that repeated administration of si‐*Ripk3*@Exo^RVG^, even over 15 consecutive dosing cycles, did not induce cytotoxicity, necrosis, or apoptosis in major organs. Collectively, these results robustly demonstrate an exceptional in vivo biocompatibility and safety profile for si‐*Ripk3*@Exo^RVG^, strongly supporting its clinical potential as a chronic therapeutic strategy for AD.

### 2.8 Si‐*Ripk3*@Exo^RVG^ Alleviates Necroptosis and Attenuates pTau‐Associated Neurotoxicity in FAD3T Mice

Following the completion of behavioral assessments, mice were euthanized, and brain tissues were harvested to systematically evaluate neuronal survival and the contribution of necroptosis in AD pathology. Immunofluorescence analysis demonstrated that FAD3T mice treated with si‐*Ripk3*@Exo^RVG^ displayed a markedly higher density of NeuN‐positive neurons in both the hippocampus and cortex compared with vehicle‐treated controls (Figure 5C,D), indicating robust neuroprotection against degeneration. Consistent with these neuroprotective effects, si‐*Ripk3*@Exo^RVG^ administration markedly suppressed the necroptotic signaling in vivo. Specifically, the proportion of pRIPK3‐positive cells was reduced by approximately 60%, decreasing from 28.48% to 12.19% (Figure 5E,F), while pMLKL‐positive cells diminished by about 50%, from 24.42% to 12.45% (Figure 5G,H). In parallel, expression of the postsynaptic density protein PSD95 was significantly restored (Figure 5I,J), suggesting preservation of synaptic integrity and neuronal connectivity. Together, these results validate the efficient brain‐targeted delivery of si‐*Ripk3* by Exo^RVG^ and underscore the potent neuroprotective effects achieved through inhibition of the RIPK3‐mediated necroptotic pathway in AD mice.

Furthermore, we observed that si‐*Ripk3*@Exo^RVG^ treatment significantly suppressed the activation of astrocytes in the brain, as evidenced by reduced GFAP immunoreactivity (Figure S5A). Quantitative analyses confirmed that the treatment decreased the number of branch points (Figure S5B) and the soma area (Figure S5C) of astrocytes, indicating an amelioration of neuroinflammation. Consistent with this, qualitative observation of 3D reconstructed microglia (Figure S5D) further illustrated the ameliorative effect of si‐*Ripk3*@Exo^RVG^ on microglial morphology.

Based on these findings, we further investigated the impact of si‐*Ripk3*@Exo^RVG^ on the core pathological hallmarks of AD. In addition to mitigating neuronal loss, this treatment markedly attenuated Tau pathology. Immunofluorescence staining and quantitative analyses revealed a significant reduction in tau hyperphosphorylation (Figure S6), particularly at the disease‐relevant Ser202 and Thr205 epitopes (Figure S6D). These results demonstrate that suppression of necroptosis not only promotes neuronal survival but also alleviates pTau‐associated neurotoxicity.

Collectively, these in vivo data demonstrate that engineered exosome‐mediated RIPK3 silencing simultaneously restrains necroptotic cell death and mitigates Tau pathology, thereby preserving neuronal and synaptic integrity in FAD3T mice. This study therefore provides compelling preclinical evidence supporting RIPK3 as a therapeutic target for AD and highlights the translational potential of neuron‐targeted exosome‐based precision RNA delivery platforms for neurodegenerative diseases.

### 2.9 Si‐*Ripk3*@Exo^RVG^ Reprograms the Neuronal Transcriptome to Suppress Neuroinflammation and Preserve Synaptic Function in AD

To elucidate the molecular basis for the neuroprotective effects of si‐*Ripk3*@Exo^RVG^, transcriptomic profiling was performed to systematically interrogate its impact on the neuronal gene expression network.

RNA sequencing of hippocampal tissue from FAD3T mice revealed that si‐*Ripk3*@Exo^RVG^ treatment induced a profound remodeling of the gene expression landscape. Relative to vehicle‐treated controls, a substantial proportion of dysregulated genes exhibited partial or complete normalization following treatment (Figure S15B,C). Gene Ontology (GO) enrichment analysis further demonstrated that these differentially expressed genes were predominantly associated with synaptic organization, neuronal survival, and intracellular signal transduction (Figure S15D). Notably, genes involved in synaptic plasticity and neuronal connectivity, including *Tulp1* and *Slitrk6*, were significantly upregulated. In contrast, genes linked to cellular stress responses, inflammation and apoptosis, such as *Hmox1*, *Icam1*, *Tnf*, and *Bag3*, were significantly downregulated (Figure S15D). This coordinated shift delineates a molecular framework through which si‐*Ripk3*@Exo^RVG^ promotes neuronal resilience while suppressing degenerative signaling.

Pathway‐level interrogation using KEGG enrichment analysis further substantiated these findings. Canonical pathways implicated in neuroinflammation and neurotoxicity, including NF‐κB, TNF, IL‐17, and MAPK signaling, were significantly attenuated following si‐*Ripk3*@Exo^RVG^ treatment (Figure S15E). Among these, MAPK signaling is of particular relevance, as it integrates inflammatory cascades with neuronal apoptosis, tau hyperphosphorylation, and neurofibrillary tangle formation. The concerted downregulation of this pathway therefore provides compelling transcriptomic evidence that RIPK3 silencing indirectly mitigates tau pathology and Aβ‐associated neurotoxicity. Conversely, genes involved in synaptic transmission and neuronal excitability, such as *Adra2b*, *Kcnmb3*, *Gnb3*, *Slc6a12*, and *Slc6a13*, were significantly upregulated. Enrichment analysis linked these changes to activation of the cGMP–PKG signaling pathway, GABAergic synapse, and synaptic vesicle cycle (Figure S15F), offering a mechanistic explanation for the observed improvements in synaptic plasticity and cognitive performance. Furthermore, the enrichment of the tyrosine metabolism and tryptophan metabolism pathways was consistent with restored neurometabolic homeostasis, further supporting the functional relevance of these transcriptional changes.

To assess regional robustness, transcriptomic analyses were extended to the cerebral cortex. Consistent with hippocampal findings, si‐*Ripk3*@Exo^RVG^ treatment induced substantial transcriptomic reorganization (Figure S7A,B), characterized by suppression of inflammatory and neurotoxic pathways such as NF‐κB, TNF, and MAPK, while activating key pathways including axon guidance and GABAergic synapses (Figure S7C–E). This confirms that si‐*Ripk3*@Exo^RVG^ can systematically exert coordinated anti‐inflammatory, pro‐survival, and synapse‐enhancing protective effects across multiple brain regions vulnerable in AD.

To validate whether these transcriptomic alterations translated into functional signaling modulation, targeted protein‐level analyses were performed. Western blotting confirmed that si‐*Ripk3*@Exo^RVG^ treatment significantly reduced phosphorylation of p38 MAPK (Figure S16A), consistent with dampened inflammatory and stress signaling. In parallel, phosphorylation of VASP, a downstream effector of the cGMP‐PKG pathway, was also suppressed (Figure S16C), corroborating the transcriptomic activation of synapse‐associated signaling cascades. These protein‐level validations establish a direct mechanistic link between transcriptional reprogramming and functional pathway modulation.

Collectively, transcriptomic profiling of the hippocampus and cortex demonstrates that si‐*Ripk3*@Exo^RVG^ drives a profound molecular reprogramming, directly targeting the core pathological networks underlying AD. This strategy achieves this by synergistically suppressing neurotoxic pathways (e.g., MAPK and NF‐κB) and activating neuroprotective programs (e.g., cGMP‐PKG and GABAergic signaling); this strategy restores transcriptional balance across affected brain regions (Figure S16D). The convergence of transcriptomic and protein‐level evidence underscores the mechanistic robustness of RIPK3‐targeted intervention and highlights the broad therapeutic potential of neuron‐targeted exosomal RNA delivery for neurodegenerative disease modulation.

### 2.10 Verification of Exo@si‐*Ripk3* Efficacy Based on Human Cortical Organoids

To bridge the gap between basic research and translational relevance, human pluripotent stem cells (hPSCs)‐derived cortical organoids were generated using an optimized protocol (Figure S8A). Immunofluorescence characterization confirmed that these organoids possess complete lamination structures, apicobasal polarity, and an active proliferating population of neural stem cells, thereby partially mimicking in vivo cortical stratification (Figures S8B–D and S9A,B). Moreover, the formation of dense neural networks and the generation of mature astrocytes indicate neuron‐glia co‐development (Figure S9C,D), providing a structurally and cellularly relevant platform for modeling cortical microenvironments and evaluating therapeutic efficacy.

**FIGURE 6:**
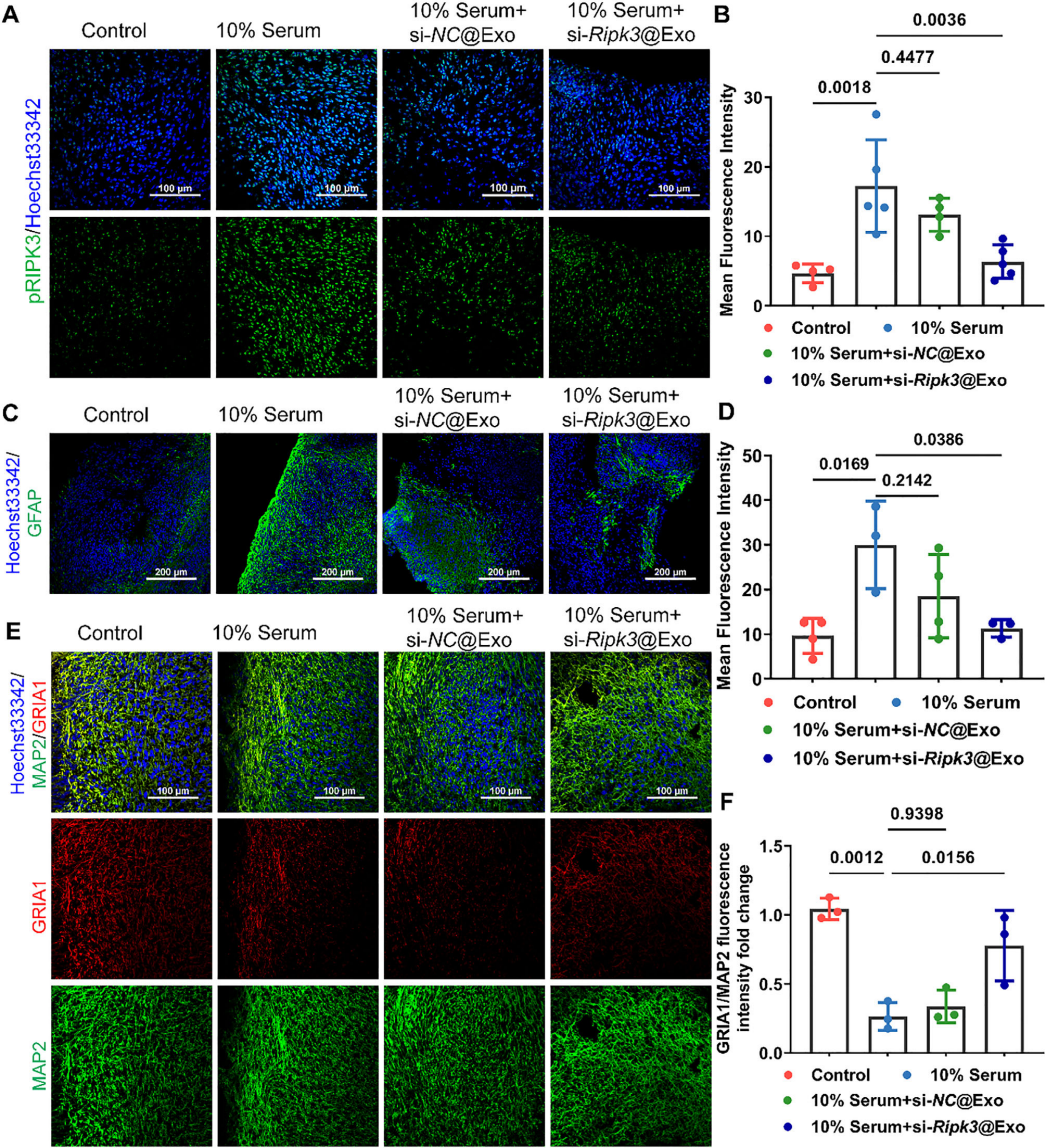
Si‐*Ripk3*@Exo treatment attenuates human serum‐induced necroptosis in cortical organoids, thereby alleviating gliosis and synaptic dysfunction. (A) Representative image of pRIPK3(Ser232) immunofluorescence. (B) Mean fluorescence intensity of pRIPK3 in ROI. (C) Representative image of GFAP immunofluorescence. (D) Mean fluorescence intensity of GFAP in ROI. (E) Representative image of co‐staining for MAP2 and GRIA1 immunofluorescence. (F) Fold change in GRIA1 fluorescence intensity is expressed as theGRIA1/MAP2 ratio and normalized to the control. Statistical analysis was performed using one‐way ANOVA followed by Tukey's post hoc test, and data points represent data from individual organoids, *n* ≥ 3 (from 3 independent differentiations).

A sporadic AD‐like model was subsequently established through 10% normal human serum treatment, which reproducibly induced hallmark pathological features, including Tau hyperphosphorylation, Aβ accumulation, activation of necroptosis pathways and neuroinflammation. For exosome‐based intervention in cortical organoids, a stable HEK293T‐RVG cell line was constructed. This line exhibited robust LAMP2 expression and secreted exosomes with a characteristic diameter of approximately 100 nm (Figure S10A,B). Importantly, nanoparticle tracking analysis revealed no significant change in exosome size distribution following electroporation, indicating preservation of vesicle integrity (Figure S10C). In addition, the si‐*Ripk3*‐1352 sequence (Figure S11) with a silencing efficiency of about 70% was screened for subsequent experiments. Next, treatment with si‐*Ripk3*@Exo markedly attenuated serum‐induced molecular and cellular damage in cortical organoids. Specifically, si‐*Ripk3*@Exo effectively suppressed necroptosis, as evidenced by reduced pRIPK3 signaling (Figure 6A,B; Figure S17E,F), and significantly alleviated reactive gliosis (Figure 6C,D). Although si‐*Ripk3*@Exo exhibited an inhibitory trend on serum‐induced AD‐like pathologies (Figure S17A–D), western blot analysis indicated that the reduction in p‐Tau levels did not reach statistical significance. Importantly, functional assessment revealed a significant restoration of the synaptic protein GRIA1 (Figure 6E,F), indicating that si‐*Ripk3*@Exo not only mitigated molecular pathology but also rescued synaptic integrity at the cellular functional level. Taken together, these results establish a robust mechanistic framework demonstrating that si‐*Ripk3*@Exo^RVG^ mediated RIPK3 silencing can reprogram the pathological neuronal microenvironment in AD‐like cortical organoids, shifting it from degenerative signaling toward functional homeostasis. This network‐level intervention highlights the translational potential of exosome‐based RNA therapeutics as a strategy for restoring neuronal stability in AD.

## 3 Discussion

Neurodegeneration in AD is increasingly recognized as a consequence of maladaptive cell death programs intertwined with chronic neuroinflammation. In the study, we identify and confirm that RIPK3‐mediated necroptosis acts as a central execution node linking neuronal loss to inflammatory amplification and demonstrate that neuron‐targeted RNA interference can effectively interrupt this pathological circuitry. By integrating evidence from human post‐mortem bioinformatics analysis, human cortical organoids and transgenic mouse models, our findings converge on a unified mechanism in which necroptotic neurons actively shape a hostile microenvironment that accelerates synaptic failure and disease progression.

Necroptosis represents a particularly tractable therapeutic target in AD because of its intrinsically pro‐inflammatory nature [8, 9, 35, 36, 37]. Unlike apoptosis, which is largely immunologically silent, necroptosis culminates in membrane rupture and the release of damage‐associated molecular patterns, including HMGB1 and ATP [38, 39], thereby initiating and sustaining microglial activation, which in turn exacerbates tau hyperphosphorylation and neuronal stress [12]. Although RIPK1 has previously been implicated in AD pathology, its kinase‐independent scaffolding functions complicate pharmacological inhibition and raise concerns regarding interference with essential survival signaling [13, 40]. In contrast, RIPK3 occupies a more restricted position downstream in the necroptotic cascade, making it a mechanistically precise target for intervention. Our data support the notion that selective RIPK3 suppression effectively arrests necroptotic execution while preserving upstream signaling integrity. Notably, we observed partial nuclear co‐localization of pRIPK3, which aligns with previous evidence that RIPK3 undergoes nuclear phosphorylation during early necroptosis and subsequently translocates to the cytoplasm to propagate the death signal [41, 42]. Especially, RIPK3 silencing induces a broader reprogramming of the disease‐associated transcriptional landscape. Transcriptomic analyses revealed coordinated downregulation of inflammatory and neurotoxic pathways, including MAPK and NF‐κB signaling, alongside activation of synapse‐supportive and neuromodulatory programs such as the cGMP–PKG axis. Importantly, the observed attenuation of tau hyperphosphorylation appears to arise indirectly from this microenvironmental normalization rather than from direct interference with tau‐modifying enzymes. This distinction underscores a key conceptual advance: targeting necroptosis reshapes the neuronal ecosystem at a systems level, increasing network resilience to multiple downstream pathological insults rather than addressing a single molecular lesion.

From a translational perspective, the study addresses a critical unmet need in the current AD therapeutic landscape. While recent advances in anti‐amyloid antibodies (e.g., Lecanemab) have shown efficacy in early‐stage AD by clearing plaques [1, 43, 44, 45], they offer limited benefit for moderate‐to‐severe stages where neuronal loss is already extensive and irreversible [46, 47, 48, 49, 50]. Our strategy fills this gap by providing robust neuroprotection even in the presence of established pathology, effectively slowing cognitive decline in advanced models [51]. The unique value of this study is further bolstered by the validation in human PSCs‐derived cortical organoids, which recapitulate patient‐specific pathophysiology and serve as a predictive bridge between rodent models and clinical trials [52, 53, 54]. The validation of efficacy in these organoids suggests that the therapeutic potential of si‐*Ripk3*@Exo^RVG^ may be effectively translated to human neural tissue.

Equally central to this study is the development of a delivery strategy capable of overcoming the longstanding barriers to RNA therapeutics in the central nervous system. Unlike viral vectors or synthetic nanoparticles, the developed RVG‐engineered exosomes leverage native biological pathways to cross the BBB and achieve neuron‐specific uptake [55, 56]. Although in vivo imaging reveals significant brain enrichment, we acknowledge its dimensional incompatibility with absolute biodistribution (%ID/g) due to optical attenuation [57]. Nevertheless, RVG‐exosomes exhibit time‐dependent signal enhancement, demonstrating superior accumulation kinetics to the negligible brain retention typical of LNPs [58]. This observable targeted enrichment validates the platform's efficacy in overcoming the BBB. Furthermore, the therapeutic efficacy stems from a synergistic mechanism. While naïve exosomes offer intrinsic neuroprotection [59], only si‐*Ripk3*@Exo^RVG^, combining the intrinsic benefit with potent gene silencing, fully rescued neurotoxicity in vitro. We confirmed that this synergy is enabled by efficient endosomal escape, ensuring the siRNA payload reaches the cytoplasm to silence RIPK3 and shift the cell state from pro‐death to survival.

Despite these promising outcomes, several limitations warrant careful consideration. Human cortical organoids lack vascularization and systemic immune interactions, which may influence pharmacokinetics and long‐term inflammatory responses. In addition, although repeated exosome administration was well tolerated in rodent models, the potential for cumulative immunogenicity or subtle off‐target effects warrants further investigation. Furthermore, the lack of comprehensive omics characterization (proteomics and lipidomics) of the engineered EVs represents a limitation; while their endogenous complexity raises theoretical concerns regarding safety and reproducibility, the current lack of standardized lipidomic databases and functional baselines makes comprehensive cargo interpretation challenging. A limitation of this study is the lack of single‐cell co‐localization analysis for necroptotic markers. Future studies employing single‐cell omics or multiplexed imaging are warranted to definitively map the cell‐type‐specific modulation of the RIPK3 pathway and further refine the targeting specificity of this therapeutic strategy. Dedicated brain imaging with optimized exposure will further enhance the quantitative sensitivity of brain accumulation, representing a valuable direction for future evaluation. Future studies in non‐human primates will be essential to evaluate chronic safety, biodistribution, and dosing paradigms, as well as to explore combinatorial strategies integrating necroptosis inhibition with upstream disease‐modifying therapies, such as anti‐amyloid antibodies, to simultaneously target upstream etiology and downstream neurodegeneration [44, 60]. Additionally, evolving multi‐omics technologies will be required to establish clearer structure‐activity relationships for EV‐based therapeutics.

## 4 Conclusion

In conclusion, this study establishes RIPK3‐mediated necroptosis as a central mechanistic bridge between neuronal loss and neuroinflammation in AD. And it introduces a neuron‐targeted exosomal RNA delivery platform (si‐*Ripk3*@Exo^RVG^) capable of modulating this pathway in vivo. By coupling precise molecular intervention with advanced biological delivery, this work highlights a viable strategy for reprogramming the degenerating neuronal microenvironment and offers a translational framework for RNA‐based therapeutics in advanced neurodegenerative disease.

## 5 Experimental Section/Methods

Detailed methods are described in the Supporting Information.

This study is a basic research involving in vitro cell experiments and animal model studies and does not involve clinical trials. Data are available upon request from the corresponding author. This study is not subject to clinical trial registration requirements, and thus no clinical trial registration number is required.

## Author Contributions

F.C. and C.Z. conceived the project and designed the study. C.Z. performed the experiments, analyzed the data, and wrote the original draft. J.Z. assisted in performing the experiments and refined the study design. J.Z., P.L., Y.W., and Y.W. conducted the animal experiments and assisted with data interpretation. Y.W. and Z.T. performed the physicochemical characterization experiments. F.C. and Q.Z. supervised the project and provided critical resources. F.C., J.Y., and Q.Z. reviewed and edited the manuscript. F.C. supervised the conduct of the study. All authors discussed the results and commented on the manuscript.

## Funding

The work was supported by the National Key R&D Program of China (2023YFA1801900), the National Natural Science Foundation of China (82572678), the Natural Science Foundation of Heilongjiang Province (Excellent Young Scholars Program, JJ2024YX0560), the “Spring Goose” Talent Team Support Program of Heilongjiang Province (2022CY CX0202), the Funding Program for Preferential Returned Scholars of Heilongjiang Province (21032240006), and the Young Elite Scientist Sponsorship Program of Heilongjiang Province (20240NTJ 018).

## Conflicts of Interest

The authors declare no conflicts of interest.

## Supporting information




**Supporting File 1**: advs76558‐sup‐0001‐SuppMat.docx.

**Supporting File 2**: advs76558sup‐0002‐DataFile.xls.

## Data Availability

The data that supports the findings of this study are available in the supplementary material of this article.

## References

[1] S. Ostrowitzki , T. Bittner , K. M. Sink , S. Ostrowitzki , T. Bittner , and K. M. Sink , “Evaluating the Safety and Efficacy of Crenezumab vs Placebo in Adults with Early Alzheimer Disease,” JAMA Neurology 79 (2022): 1113, 10.1001/jamaneurol.2022.2909. 36121669 PMC9486635

[2] X. Q. Chen , A. Salehi , M. L. Pearn , X. Q. Chen , A. Salehi , and M. L. Pearn , “Targeting Increased Levels of APP in Down syndrome: Posiphen‐Mediated Reductions in APP and Its Products Reverse Endosomal Phenotypes in the Ts65Dn Mouse Model,” Alzheimer's & Dementia 17 (2021): 271–292, 10.1002/alz.12185. PMC7984396 32975365

[3] K. Pang , R. Jiang , W. Zhang , K. Pang , R. Jiang , and W. Zhang , “An App Knock‐In Rat Model for Alzheimer's Disease Exhibiting Aβ and Tau Pathologies, Neuronal Death and Cognitive Impairments,” Cell Research 32 (2022): 157–175, 10.1038/s41422-021-00582-x. 34789895 PMC8807612

[4] C. R. Jack Jr. , J. S. Andrews , T. G. Beach , C. R. Jack Jr. , J. S. Andrews , and T. G. Beach , “Revised Criteria for Diagnosis and Staging of Alzheimer's Disease: Alzheimer's Association Workgroup,” Alzheimer's & Dementia 20 (2024): 5143–5169, 10.1002/alz.13859. PMC11350039 38934362

[5] S. Balusu , K. Horré , N. Thrupp , S. Balusu , K. Horré , and N. Thrupp , “MEG3 Activates Necroptosis in human Neuron Xenografts Modeling Alzheimer's Disease,” Science 381 (2023): 1176–1182, 10.1126/science.abp9556. 37708272 PMC7615236

[6] M. J. Koper , E. Van Schoor , S. Ospitalieri , M. J. Koper , E. Van Schoor , and S. Ospitalieri , “Necrosome Complex Detected in Granulovacuolar Degeneration Is Associated with Neuronal Loss in Alzheimer's Disease,” Acta Neuropathologica 139 (2020): 463–484, 10.1007/s00401-019-02103-y. 31802237

[7] S. Zhou , W. Zhang , G. Cai , S. Zhou , W. Zhang , and G. Cai , “Myofiber Necroptosis Promotes Muscle Stem Cell Proliferation via Releasing Tenascin‐C during Regeneration,” Cell Research 30 (2020): 1063–1077, 10.1038/s41422-020-00393-6. 32839552 PMC7784988

[8] X. Li , C. Q. Zhong , R. Wu , X. Li , C. Q. Zhong , and R. Wu , “RIP1‐Dependent Linear and Nonlinear Recruitments of Caspase‐8 and RIP3 Respectively to Necrosome Specify Distinct Cell Death Outcomes,” Protein & Cell 12 (2021): 858–876, 10.1007/s13238-020-00810-x. 33389663 PMC8563874

[9] K. Newton , A. Strasser , N. Kayagaki , et al., “Cell Death,” Cell 187 (2024): 235–256, 10.1016/j.cell.2023.11.044. 38242081

[10] H. Meng , G. Wu , X. Zhao , H. Meng , G. Wu , and X. Zhao , “Discovery of a Cooperative Mode of Inhibiting RIPK1 Kinase,” Cell Discovery 7 (2021): 41, 10.1038/s41421-021-00278-x. 34075030 PMC8169668

[11] M. J. Koper , S. Moonen , A. Ronisz , M. J. Koper , S. Moonen , and A. Ronisz , “Inhibition of an Alzheimer's Disease–associated Form of Necroptosis Rescues Neuronal Death in Mouse Models,” Science Translational Medicine 16 (2024): adf5128, 10.1126/scitranslmed.adf5128. 39475569

[12] M. Kamiya , F. Mizoguchi , K. Kawahata , M. Kamiya , F. Mizoguchi , and K. Kawahata , “Targeting Necroptosis in Muscle Fibers Ameliorates Inflammatory Myopathies,” Nature Communications 13 (2022): 166, 10.1038/s41467-021-27875-4. PMC8748624 35013338

[13] H. Yin , X. Guo , Y. Chen , H. Yin , X. Guo , and Y. Chen , “TAB2 deficiency Induces Dilated Cardiomyopathy by Promoting RIPK1‐Dependent Apoptosis and Necroptosis,” Journal of Clinical Investigation 132 (2022): 152297, 10.1172/jci152297. PMC8843707 34990405

[14] X. Li , F. Li , X. Zhang , X. Li , F. Li , and X. Zhang , “Caspase‐8 Auto‐Cleavage Regulates Programmed Cell Death and Collaborates with RIPK3/MLKL to Prevent Lymphopenia,” Cell Death & Differentiation 29 (2022): 1500–1512, 10.1038/s41418-022-00938-9. 35064213 PMC9345959

[15] T. G. Nguyen Cao , J. H. Kang , W. Kim , T. G. Nguyen Cao , J. H. Kang , and W. Kim , “Engineered Extracellular Vesicle‐Based Sonotheranostics for Dual Stimuli‐Sensitive Drug Release and Photoacoustic Imaging‐Guided Chemo‐Sonodynamic Cancer Therapy,” Theranostics 12 (2022): 1247–1266, 10.7150/thno.65516. 35154485 PMC8771566

[16] A. C. Yang , R. T. Vest , F. Kern , A. C. Yang , R. T. Vest , and F. Kern , “A Human Brain Vascular Atlas Reveals Diverse Mediators of Alzheimer's Risk,” Nature 603 (2022): 885–892, 10.1038/s41586-021-04369-3. 35165441 PMC9635042

[17] A. Arguello , C. S. Mahon , M. E. K. Calvert , A. Arguello , C. S. Mahon , and M. E. K. Calvert , “Molecular Architecture Determines Brain Delivery of a Transferrin Receptor–targeted Lysosomal Enzyme,” Journal of Experimental Medicine 219 (2022): 20211057, 10.1084/jem.20211057. PMC8932535 35226042

[18] J. A. Kulkarni , D. Witzigmann , S. B. Thomson , J. A. Kulkarni , D. Witzigmann , and S. B. Thomson , “The Current Landscape of Nucleic Acid Therapeutics,” Nature Nanotechnology 16 (2021): 630–643, 10.1038/s41565-021-00898-0. 34059811

[19] L. Zhang , Z. Qin , H. Sun , L. Zhang , Z. Qin , and H. Sun , “Nanoenzyme Engineered Neutrophil‐Derived Exosomes Attenuate Joint Injury in Advanced Rheumatoid Arthritis via Regulating Inflammatory Environment,” Bioactive Materials 18 (2022): 1–4, 10.1016/j.bioactmat.2022.02.017. 35387158 PMC8961303

[20] S. Hu , Z. Li , D. Shen , S. Hu , Z. Li , and D. Shen , “Exosome‐Eluting Stents for Vascular Healing after Ischaemic Injury,” Nature Biomedical Engineering 5 (2021): 1174–1188, 10.1038/s41551-021-00705-0. PMC8490494 33820981

[21] Y. Lin , M. Yan , Z. Bai , Y. Lin , M. Yan , and Z. Bai , “Huc‐MSC‐Derived Exosomes Modified with the Targeting Peptide of aHSCs for Liver Fibrosis Therapy,” Journal of Nanobiotechnology 20 (2022): 432, 10.1186/s12951-022-01636-x. 36183106 PMC9526331

[22] X. Ma , M. Yao , Y. Gao , X. Ma , M. Yao , and Y. Gao , “Functional Immune Cell‐Derived Exosomes Engineered for the Trilogy of Radiotherapy Sensitization,” Advanced Science 9 (2022): 2106031, 10.1002/advs.202106031. 35715382 PMC9376809

[23] J. Han , J. H. Sul , J. Lee , J. Han , J. H. Sul , and J. Lee , “Engineered Exosomes with a Photoinducible Protein Delivery System Enable CRISPR‐Cas–based Epigenome Editing in Alzheimer's Disease,” Science Translational Medicine 16 (2024): adi4830, 10.1126/scitranslmed.adi4830. 39110781

[24] Y. Zhuo , Z. Luo , Z. Zhu , Y. Zhuo , Z. Luo , and Z. Zhu , “Direct Cytosolic Delivery of siRNA via Cell Membrane Fusion Using Cholesterol‐Enriched Exosomes,” Nature Nanotechnology 19 (2024): 1858–1868, 10.1038/s41565-024-01785-0. 39300226

[25] M. Ma , J. Wang , W. Zhong , et al., “Cleavable Antibody‐Conjugated Aβ Specific Immune Exosome for Combination Alzheimer's Disease Immunotherapy,” Angewandte Chemie International Edition 64 (2025): 202517917, 10.1002/anie.202517917. 41013939

[26] Z. Li , J. Yang , J. Li , Z. Li , J. Yang , and J. Li , “Targeted Delivery of BACE1 siRNA for Synergistic Treatment of Alzheimer's Disease,” Translational Neurodegeneration 14 (2025): 41, 10.1186/s40035-025-00503-7. 40814010 PMC12351871

[27] A. Caccamo , C. Branca , I. S. Piras , A. Caccamo , C. Branca , and I. S. Piras , “Necroptosis Activation in Alzheimer's Disease,” Nature Neuroscience 20 (2017): 1236–1246, 10.1038/nn.4608. 28758999

[28] D. Ha , N. Yang , V. Nadithe , D. Ha , N. Yang , and V. Nadithe , “Exosomes as Therapeutic Drug Carriers and Delivery Vehicles across Biological Membranes: Current Perspectives and Future Challenges,” Acta Pharmaceutica Sinica B 6 (2016): 287–296, 10.1016/j.apsb.2016.02.001. 27471669 PMC4951582

[29] A. Iyaswamy , A. Thakur , X.‐J. Guan , A. Iyaswamy , A. Thakur , and X.‐J. Guan , “Fe65‐Engineered Neuronal Exosomes Encapsulating Corynoxine‐B Ameliorate Cognition and Pathology of Alzheimer's Disease,” Signal Transduction and Targeted Therapy 8 (2023): 404, 10.1038/s41392-023-01657-4. 37867176 PMC10590775

[30] J. Bolik , F. Krause , M. Stevanovic , J. Bolik , F. Krause , and M. Stevanovic , “Inhibition of ADAM17 Impairs Endothelial Cell Necroptosis and Blocks Metastasis,” Journal of Experimental Medicine 219 (2022): 20201039, 10.1084/jem.20201039. PMC8689681 34919140

[31] Z. Liu , Y. Xu , Y. Wan , Z. Liu , Y. Xu , and Y. Wan , “Exosomes from Adipose‐Derived Mesenchymal Stem Cells Prevent Cardiomyocyte Apoptosis Induced by Oxidative Stress,” Cell Death Discovery 5 (2019): 79, 10.1038/s41420-019-0159-5. 30911413 PMC6425027

[32] Y. Xu , Y. Du , Q. Hu , Y. Xu , Y. Du , and Q. Hu , “Mesenchymal Stem Cell‐Derived Exosomes Alleviate Oxidative Stress and Brain Injuries through Promoting OPA1 Mediated Mitochondrial Fusion after Intracerebral Hemorrhage,” Molecular Neurobiology 63 (2026): 426, 10.1007/s12035-026-05703-4. 41652113 PMC12881117

[33] J. J. Liu , R. W. Tsien , Z. P. Pang , J. J. Liu , R. W. Tsien , and Z. P. Pang , “Hypothalamic Melanin‐Concentrating Hormone Regulates Hippocampus‐Dorsolateral Septum Activity,” Nature Neuroscience 25 (2022): 61–71, 10.1038/s41593-021-00984-5. 34980924 PMC8741735

[34] L. Yang , C. Wu , E. Parker , L. Yang , C. Wu , and E. Parker , “Non‐Invasive Photobiomodulation Treatment in an Alzheimer Disease‐Like Transgenic Rat Model,” Theranostics 12 (2022): 2205–2231, 10.7150/thno.70756. 35265207 PMC8899582

[35] Q. Chen , K. Ma , X. Liu , Q. Chen , K. Ma , and X. Liu , “Truncated PARP1 Mediates ADP‐Ribosylation of RNA Polymerase III for Apoptosis,” Cell Discovery 8 (2022): 3, 10.1038/s41421-021-00355-1. 35039483 PMC8764063

[36] A. G. Johnson , T. Wein , M. L. Mayer , A. G. Johnson , T. Wein , and M. L. Mayer , “Bacterial Gasdermins Reveal an Ancient Mechanism of Cell Death,” Science 375 (2022): 221–225, 10.1126/science.abj8432. 35025633 PMC9134750

[37] X. Wu , K. L. Poulsen , C. Sanz‐Garcia , X. Wu , K. L. Poulsen , and C. Sanz‐Garcia , “MLKL‐Dependent Signaling Regulates Autophagic Flux in a Murine Model of Non‐Alcohol‐Associated Fatty Liver and Steatohepatitis,” Journal of Hepatology 73 (2020): 616–627, 10.1016/j.jhep.2020.03.023. 32220583 PMC7438259

[38] C. Chen , Z. Wang , S. Jia , C. Chen , Z. Wang , and S. Jia , “Evoking Highly Immunogenic Ferroptosis Aided by Intramolecular Motion‐Induced Photo‐Hyperthermia for Cancer Therapy,” Advanced Science 9 (2022): 2104885, 10.1002/advs.202104885. 35132824 PMC8981454

[39] S. W. Tse , K. McKinney , W. Walker , S. W. Tse , K. McKinney , and W. Walker , “mRNA‐Encoded, Constitutively Active STINGV155M Is a Potent Genetic Adjuvant of Antigen‐Specific CD8+ T Cell Response,” Molecular Therapy 29 (2021): 2227–2238, 10.1016/j.ymthe.2021.03.002. 33677092 PMC8261085

[40] T. Riebeling , K. Jamal , R. Wilson , T. Riebeling , K. Jamal , and R. Wilson , “Primidone Blocks RIPK1‐Driven Cell Death and Inflammation,” Cell Death & Differentiation 28 (2021): 1610–1626, 10.1038/s41418-020-00690-y. 33273695 PMC7712602

[41] Y. Yang , J. Ma , Y. Chen , et al., “Nucleocytoplasmic Shuttling of Receptor‐Interacting Protein 3 (RIP3),” Journal of Biological Chemistry 279 (2004): 38820–38829, 10.1074/jbc.M401663200. 15208320

[42] K. Weber , R. Roelandt , I. Bruggeman , et al., “Nuclear RIPK3 and MLKL Contribute to Cytosolic Necrosome Formation and Necroptosis,” Communications Biology 1 (2018): 6, 10.1038/s42003-017-0007-1. 30271893 PMC6123744

[43] R. J. Bateman , J. Smith , M. C. Donohue , R. J. Bateman , J. Smith , and M. C. Donohue , “Two Phase 3 Trials of Gantenerumab in Early Alzheimer's Disease,” New England Journal of Medicine 389 (2023): 1862–1876, 10.1056/NEJMoa2304430. 37966285 PMC10794000

[44] C. H. van Dyck , C. J. Swanson , P. Aisen , C. H. van Dyck , C. J. Swanson , and P. Aisen , “Lecanemab in Early Alzheimer's Disease,” New England Journal of Medicine 388 (2023): 9, 10.1056/NEJMoa2212948. 36449413

[45] J. R. Sims , J. A. Zimmer , C. D. Evans , J. R. Sims , J. A. Zimmer , and C. D. Evans , “Donanemab in Early Symptomatic Alzheimer Disease,” Jama 330 (2023): 512, 10.1001/jama.2023.13239. 38112817

[46] L. Traxler , J. R. Herdy , D. Stefanoni , L. Traxler , J. R. Herdy , and D. Stefanoni , “Warburg‐Like Metabolic Transformation Underlies Neuronal Degeneration in Sporadic Alzheimer's Disease,” Cell Metabolism 34 (2022): 1248–1263.e6, 10.1016/j.cmet.2022.07.014. 35987203 PMC9458870

[47] A. Disouky , M. A. Sanborn , K. R. Sabitha , A. Disouky , M. A. Sanborn , and K. R. Sabitha , “Human Hippocampal Neurogenesis in Adulthood, Ageing and Alzheimer's Disease,” Nature 652 (2026): 1264–1273, 10.1038/s41586-026-10169-4. 41741649 PMC13048220

[48] J. Travis and J. Travis , “Latest Alzheimer's Antibody Is ‘Not a Miracle Drug’,” Science 380 (2023): 571, 10.1126/science.adi6515. 37167375

[49] R. Mishra , T. Phan , P. Kumar , R. Mishra , T. Phan , and P. Kumar , “Augmenting Neurogenesis Rescues Memory Impairments in Alzheimer's Disease by Restoring the Memory‐Storing Neurons,” Journal of Experimental Medicine 219 (2022): 20220391, 10.1084/jem.20220391. PMC9399756 35984475

[50] Y. Zhou , Y. Su , S. Li , Y. Zhou , Y. Su , and S. Li , “Molecular Landscapes of human Hippocampal Immature Neurons across Lifespan,” Nature 607 (2022): 527–533, 10.1038/s41586-022-04912-w. 35794479 PMC9316413

[51] K. Chaubey , E. Vázquez‐Rosa , S. J. Tripathi , K. Chaubey , E. Vázquez‐Rosa , and S. J. Tripathi , “Pharmacologic Reversal of Advanced Alzheimer's Disease in Mice and Identification of Potential Therapeutic Nodes in human Brain,” Cell Reports Medicine 7 (2026): 102535, 10.1016/j.xcrm.2025.102535. 41435831 PMC12866132

[52] M. A. Lancaster , J. A. Knoblich , M. A. Lancaster , and J. A. Knoblich , “Generation of Cerebral Organoids from human Pluripotent Stem Cells,” Nature Protocols 9 (2014): 2329–2340, 10.1038/nprot.2014.158. 25188634 PMC4160653

[53] S. Jiang , H. Li , L. Zhang , S. Jiang , H. Li , and L. Zhang , “Generic Diagramming Platform (GDP): A Comprehensive Database of High‐Quality Biomedical Graphics,” Nucleic Acids Res 53 (2025): D1670, 10.1093/nar/gkae973. 39470721 PMC11701665

[54] D. Sun , X. Guan , A. E. Moran , D. Sun , X. Guan , and A. E. Moran , “Identifying Phenotype‐Associated Subpopulations by Integrating Bulk and Single‐Cell Sequencing Data,” Nature Biotechnology 40 (2022): 527–538, 10.1038/s41587-021-01091-3. PMC9010342 34764492

[55] Z. Yang , J. Shi , J. Xie , Z. Yang , J. Shi , and J. Xie , “Large‐Scale Generation of Functional mRNA‐Encapsulating Exosomes via Cellular Nanoporation,” Nature Biomedical Engineering 4 (2020): 69–83, 10.1038/s41551-019-0485-1. PMC7080209 31844155

[56] B. Li , X. Chen , W. Qiu , B. Li , X. Chen , and W. Qiu , “Synchronous Disintegration of Ferroptosis Defense Axis via Engineered Exosome‐Conjugated Magnetic Nanoparticles for Glioblastoma Therapy,” Advanced Science 9 (2022): 2105451, 10.1002/advs.202105451. 35508804 PMC9189685

[57] V. Ntziachristos , J. Ripoll , L. V. Wang , et al., “Looking and Listening to Light: The Evolution of Whole‐Body Photonic Imaging,” Nature Biotechnology 23 (2005): 313–320, 10.1038/nbt1074. 15765087

[58] X. Hou , T. Zaks , R. Langer , et al., “Lipid Nanoparticles for mRNA Delivery,” Nature Reviews Materials 6 (2021): 1078–1094, 10.1038/s41578-021-00358-0. 34394960 PMC8353930

[59] A. Singh , A. Raghav , P. A. Shiekh , et al., “Transplantation of Engineered Exosomes Derived from Bone Marrow Mesenchymal Stromal Cells Ameliorate Diabetic Peripheral Neuropathy under Electrical Stimulation,” Bioactive Materials 6 (2021): 2231–2249, 10.1016/j.bioactmat.2021.01.008. 33553812 PMC7829156

[60] I. Terao , W. Kodama , I. Terao , and W. Kodama , “Comparative Efficacy, Tolerability and Acceptability of Donanemab, lecanemab, Aducanumab and Lithium on Cognitive Function in Mild Cognitive Impairment and Alzheimer's Disease: A Systematic Review and Network Meta‐Analysis,” Ageing Research Reviews 94 (2024): 102203, 10.1016/j.arr.2024.102203. 38253184

