# Supplementary material for: Neuron‐Targeted Exosomal Delivery of siRNA Against RIPK3 Slows Neurodegenerative Progression in Alzheimer's Disease
Source: Adv Sci (Weinh). 2026 Jul 11;13(55):e76558. doi: 10.1002/advs.76558 (PMC13355932; doi:10.1002/advs.76558)
Supplement: Supplementary file 1 [file ADVS-13-e76558-s001.docx]

Supporting Information

Neuron-targeted exosomal delivery of siRNA against RIPK3 slows neurodegenerative progression in Alzheimer’s disease

*Chi Zhang ^†^, Jiaqi Zhang^†^, Yuzhi Wang, Yaodong Wang, Peng Lin, Yanling Wang, Zixuan Tang, Jintai Yu^*^, Qin Zhou ^*^, Feiyun Cui ^*^*

**1. Materials and Methods**

**1.1 Chemical and reagents**

Goat Anti-Rabbit IgG (H+L) HRP (Cat# BS20241-Y) and Goat Anti-Mouse IgG (H+L) HRP (Cat# BS20242-Y) secondary antibodies were purchased from Bioworld Technology CO., Ltd. Dulbecco’s Modiﬁed Eagle Medium (DMEM) (Cat# 11965084, Gibco), fetal bovine serum (FBS) (Cat# CF-01P-02, Cell-Box), penicillin-streptomycin solution (Cat# C0222, Beyotime), Lipofectamine 3000 reagent (Cat# L3000015, Invitrogen), Opti-MEM medium (Cat# 31985070, Gibco), X-tremeGENE™ siRNA Transfection Reagent (Cat# 4476093001, Roche), RIPA lysis buffer (Cat# P0013B, Beyotime), protease inhibitors (Cat# ST506, Beyotime), phosphatase inhibitors (Cat# P1081, Beyotime), BCA assay reagent (Cat# Cat# P0010, Beyotime), Triton X-100 (Cat# T9284, Sigma), 10X SDS-PAGE Electrophoresis Buffer with Tris-Gly (Cat# P0014D, Beyotime), PVDF membrane (Cat# 10600023, Millipore), Western Transfer Buffer (Cat# P0021B, Beyotime), Protein ladder (Cat# WJ103, epizyme), Anti-Fading Mounting Medium for Fluorescence (Cat# S2100, Solarbio), paraformaldehyde (PFA) (Cat# P6148), ECL kit (Cat# M2301, SuperSignal, HaiGene), Cell Supernatant Exosome Extraction Kit (Cat#  C3620S, Precipitation Method，Beyotime), PKH26 Red Fluorescent Cell Linker Kits(Cat# UR52302，Umibio), Amicon® Ultra Centrifugal Filtration Tubes (Cat# UFC905008, 50 kDa MWCO, sample volume 15 mL, Merck), Amicon® Ultra Centrifugal Filtration Tubes (Cat# UFC505008，50 kDa MWCO, sample volume 0.5 mL, Merck), Okadaic acid (Cat# 78111-17-8, ≥95%(HPLC), Shanghai yuanye Bio-Technology Co., Ltd), Lyso-tracker Red(Cat# C1046, Beyotime), Cy7 DiC18(DiR, Cat# HY-D1048, MedChemExpress).

**1.2 Animals**

Animal-related experiments were conducted in accordance with the approved schemes by the Ethics Committee for Human and Animal Experiments of Harbin Medical University, which utilize human and animal experiment subjects (Review ID: HMUIRB2025038). Researchers involved in the experiments obtained approval from relevant health departments and regulations, and animal experiments licenses were obtained prior to the start of the experiments. All transgenic mouse populations were bred and maintained under standard specific-pathogen-free (SPF) laboratory animal facilities, equipped with independent ventilated cages (IVCs), ensuring consistent temperature (approximately 22 ± 2 °C), humidity (approximately 50 ± 10%), lighting cycle (12-hours on/off), and regular cleaning and disinfection of cage and bedding materials. Health and behavioral assessments of mice will be closely monitored during the experiments, adhering strictly to the Guidelines for Euthanasia of Animals: 2021 Edition published by the AVMA. FAD3T^APP/PS1/MAPT^(FAD3T) mice, along with their wild-type C57BL/6 J littermates, were purchased from GemPharmatech Co., Ltd., Nanjing, China. These mice carrying the Swedish mutation in the human APOE gene, the M146V mutation in the human PSEN1 gene, and the P301L mutation in the human MAPT gene can detect diagnostic biomarkers, pathological characteristics, and neurobehavioral phenotypes associated with Alzheimer’s disease, and their disease progression follows the clinical course of Alzheimer’s patients.

**1.3 Cell culture**

Mouse hippocampal neuronal cells (HT22), human embryonic kidney 293T cells (HEK293T), human neuroblastoma cell line (SH-SY5Y), mouse brain microvascular endothelial cells (bEnd.3), mouse brain vascular pericytes (MBVP), and mouse cerebellar astrocytes (C8-D1A) were cultured in standard DMEM medium supplemented with 10% fetal bovine serum (FBS) and 1% penicillin-streptomycin solution, and maintained in a humidified incubator at 37°C with 5% CO2.

Human embryonic stem cells (H1 and H9) were purchased from Yimo Biotechnology Co., Ltd. and cultured in a chemically defined, feeder-free, and serum-free mTeSR™1 culture system.

**1.4 Treatment of drugs based-on Exosomes**

3-month-old FAD3T^APP/PS1/TAU^ and C57BL/6J mice were randomly assigned to the WT-Vehicle, AD-Vehicle, and AD-si-*Ripk3*@Exo^RVG^ groups for the purpose of treatment planning and behavioral experiments. All drug formulations were administered intravenously (IV) to the FAD3T and C57BL/6J (wild type; WT) mouse models, with injections administered every 3 days. Mice were intravenously injected with targeted exosomes at a dose of 1 mg/kg (exosomal protein) every three days, lasting for a total of 45 days. For a 20 g mouse, this corresponds to approximately 20 μg of protein 2×10^10^particles) per injection. HT22 cells were used for most of the cell culture experiments.

**1.5 Genetically modify hippocampal neuron cells to produce RVG-engineered exosomes**

RVG-engineered exosomes were produced by infecting HT22 cells with the pLV3-CMV-RVG-Lamp2-Puro lentivirus. In short, cultured 293T cells were transiently transfected with the pLV3-CMV-RVG-Lamp2-Puro plasmid using Lipofectamine 3000 reagent for 48-72 hours, and the supernatant was collected as conditioned medium, which served as the pLV3-CMV-RVG-Lamp2-Puro lentivirus. Next, the collected lentivirus was used to infect HT22 cells for at least 48 hours, with the addition of 10μg/mL puromycin for selective pressure to obtain a stable HT22-RVG cell line that expressed RVG-Lamp2. Following this, HT22-RVG cells were cultured to the logarithmic growth phase and the culture medium was replaced with serum-free DMEM. After continuing the culture for an additional 24 hours, the supernatant was collected for the isolation of RVG-engineered exosomes, and the cells were scraped from the plate to collect cell lysate for determining the percentage of RVG-Lamp2 overexpression (OE) using immunoblot analysis.

Given that SH-SY5Y cells, as a neuroblastoma cell line, possess tumor-derived characteristics and their secreted exosomes pose potential tumorigenic risks that hinder clinical translation, this study selected HEK293T cells, which offer superior safety profiles and are commonly used for engineered exosome preparation, as the exosome donor source^1-3^. To obtain targeted human-derived exosomes, a HEK293T-RVG cell line stably expressing the RVG-LAMP2 (Human) fusion protein was constructed following the aforementioned protocol. Subsequently, exosomes secreted by this cell line were collected and applied to treat human cortical organoids.

**1.6 Isolation of exosomes**

Exosomes were isolated from the cell culture conditioned medium using the Cell Supernatant Exosome Extraction Kit. In short, the extracellular medium was collected from HT22-RVG cells cultured for 24 hours and centrifuged at 1500 × g for 5 minutes. Next, the supernatant was transferred into Amicon® Ultra Centrifugal Filtration Tubes and centrifuged at 4000×g for 15 minutes at 4°C to collect the retentate. The retentate was mixed with exosome extraction reagent at a 1/5 volume ratio and incubated overnight at 4 °C, followed by centrifugation at 10,000 × g for 30 minutes at 4 °C. The mechanism of action involves the exosome extraction reagent binding to water molecules and forcing less-soluble components such as vesicles to precipitate out of the solution, allowing them to be collected through rapid, low-speed centrifugation. The supernatant was discarded, and the pellet containing exosomes was diluted with 1 × phosphate-buffered saline (PBS) and filtered through a 0.22 μm sterile filter. The filtered exosome suspension was then subjected to particle size testing, marker protein analysis, and observation under transmission electron microscopy (TEM).

**1.7 Determining the size of exosomes by Zeta potential analyzer**

The particle size and membrane surface Zeta potential of exosomes were measured in accordance with the instructions provided by the manufacturer of the nano particle size and Zeta potential analyzer (Dandong Bettersize, China).

**1.8 Evaluation of exosomes by TEM**

The size, shape, and morphology of exosomes were identified by transmission electron microscopy (TEM) analysis. In brief, a negative staining technique was used to observe the exosomes. A drop of 30 μl of the exosome suspension in filtered PBS was placed on carbon-coated electron microscopy (CCEM) grids, set on parafilm, and incubated at room temperature for 10 minutes. Subsequently, it was transferred to a drop of Uranyless® solution for 1 minute and then left to air dry, with the excess stain being blotted away. Imaging of the exosomes processed on the CCEM grids was performed using a transmission electron microscope (Hitachi HT7700 Transmission Electron Microscope) at 80 kV.

**1.9 Loading of siRNA into exosomes and their evaluation**

The protein concentration of exosomes was measured using the BCA method. Exo (20 µg with a protein concentration of 1 µg/µl) was mixed with 1 μg of siRNA at 4°C for 30 minutes (the total volume did not exceed 200 µl). The mixture was then added to electroporation cuvettes (cap size: 2 mm). Electroporation was performed using the Gene Pulser Xcell Electroporation System (Bio-Rad, Hercules, CA, USA) with the following conditions: 350 V, 150 mA, 2 pulses. After electroporation, the exosomes were washed with normal saline and resuspended in normal saline, kept cool on ice, and immediately injected into mice. After this washing step, the dose for mice was 0.2-0.3 µg of exosome protein per injection, in a volume of 100 µl normal saline. For in vitro transfection, exosomes were electroporated and washed with PBS as described above, then treated with 200,000 cells in a 6-well plate, processed for the specified time as described for each experiment, washed with PBS, and used for further analysis. The si-*Ripk3* sequence is as follows: sense strand 5′-GGUAGACAAGACUUCACUATT-3′, antisense strand 5′-UAGUGAAGUCUUGUCUACCTT-3′, si-Ripk3-Ome, sense strand 5′-[mg]*[mg]*[mU][mA][mg][mA][2flC][mA][2flA][2flg][2flA][mC][mU][mU][mC][mA][mC][mu][mA]*dT*dT-3′, antisense strand 5′-[mU]*[2flA]*[mg][mU][mg][2flA][mA][mg][mU][mC][mU][mU][mg][2flU][mC][2flU][mA][mC][mC]*dT*dT-3′, synthesized by Shanghai Sangon. All in vivo or in vitro experiments used freshly prepared exosomes.

**1.10 Labeling and targeting in vitro of exosomes**

To measure its loading rate, the mixture after electroporation was separated through a 10 KDa MWCO ultrafiltration tube for collection, with the supernatant collected as "Wash", while the liquid in the tube after elution was recorded as "Elution". Due to the difference in particle size between exosomes and free siRNA, the free siRNA was collected in the Wash, while the siRNA loaded onto the exosomes (siRNA@Exo) was collected in the Elution. By measuring the fluorescence signal in the Elution, the corresponding content of siRNA can be calculated based on the standard curve of FAM-siRNA fluorescence. Calculate the load factor (EE (%)) by the following formulas in relation to the loaded dose (LC)：

EE (%) = n_Elution_/n_Total	(1)_

LC (mol/μg.prot Exo) = n_siRNA（elution）_/ m _Exo	(2)_

**1.11 Labeling and targeting in vitro of exosomes**

For in vitro experiments, exosomes were labeled with the PKH26 fluorescent dye (Umibio, Shanghai, China) according to the manufacturer's instructions. Briefly, PKH26 dye working solution (final concentration 5 μM) was added to the exosome solution in 100 μl of 1XPBS, mixed well, and incubated at room temperature for 10 minutes, followed by removal of free dye using Amicon® Ultra Centrifugal Filtration Tubes. HT22 cells were seeded in confocal dishes and co-cultured with PKH26-labeled Exo for 3-6 hours. Then, the cells were fixed in 4% (w/v) paraformaldehyde in PBS for 15 minutes and stained with 1.5 µg/ml 4,6-diamino-2-phenylindole dihydrochloride (DAPI, Beyotime, China) at room temperature for 5 minutes. After washing with PBS, the intracellular distribution of exosomes was observed using a fluorescence microscope (Nikon, Tokyo, Japan).

**1.12 Western blotting**

The protein concentration in cell lysates or exosomes was measured using the BCA method. Equal amounts of protein samples (about 10μg) from cells or exosomes were loaded and separated on SDS-polyacrylamide gel electrophoresis, and then transferred to a PVDF membrane for 1 hour in an ice bath to transfer all proteins. Next, the membrane containing protein samples was blocked with 5% non-fat milk in TBS-Tween 20 (0.1%) (TBST) for 2 hours at room temperature and incubated with primary antibodies overnight at 4°C. After washing with TBST three times, the membrane was incubated with HRP-conjugated secondary antibodies for 1 hour at room temperature. The desired bands were visualized using enhanced chemiluminescence reagents and a chemiluminescence imaging system (Shen Hua Technology, China).

**1.13 Analysis of behavior in AD mice**

*Nest construction.* The nest construction was conducted in accordance with established methods.^4^ The test subjects were housed in individual cages. Prior to the commencement of the test, a 1 cm thick paper pad was placed in each cage. On the first day of the test, two sheets of paper (15 cm x 15 cm, kitchen napkins) were introduced into the mice's habitat to evaluate nesting behavior. After a 24-hour period, the nest was photographed and assessed based on the following criteria: 0 points for no kitchen paper towels; 1 point for scattered paper towels throughout the cage without any noticeable bite marks (indicating active nesting); 2 points for paper towels concentrated within the cage, yet lacking obvious bite marks; 3 points for paper towels clustered in one area or corner, accompanied by some bite marks; and 4 points for the majority of the paper towels being bitten and gathered together. Paper towels that were torn into small pieces or had holes were classified as bitten. All results were scored in a blind manner.

*Open field test.* The open field test paradigm was applied to examine the locomotor and exploratory behavior of AD mice. In brief, the mice were first placed in the center of the open field, and their exploratory behavior was observed for a period of 5 minutes. After each test, the cages were routinely cleaned with ethanol. Software was used to measure the distance traveled, with center standing and wall standing as indicators of locomotor activity, while time spent in the central area, stretch attend postures, freezing, grooming, and the number of feces and urine spots were used to measure exploratory behavior and anxiety-like behavior.

*Morris Water Maze test.* All mice first underwent a straight swim pretraining protocol. Then, a hidden platform was placed in the center of one quadrant of the pool, and all mice were subjected to a 5-day reference memory training, with 4 trials per day, each lasting 120 seconds. After the last trial on the 5th day, the platform was removed from the pool. Twenty-four hours later, each mouse was tested with a single 90-second "probe trial" on the 6th day. Escape latency (in seconds), swim path, time spent in the target quadrant, and the number of platform zone crossings were recorded using an online real-time video tracking system. Behavioral data were analyzed using the corresponding software.

*Novel Object Recognition Test.* The Novel Object Recognition Test is used to assess the memory function and recognition memory of mice. Before the test, animals are given a day to familiarize themselves with the test environment, where they can freely explore the box. On the first day, the mice are trained to recognize two familiar objects placed at an equal distance from the center. On the second day, one of the objects is replaced with a novel object of a different shape. The animals' exploration behavior towards the novel object is then recorded for 5-10 minutes using an online real-time video tracking system. Software is used to calculate the percentage of recognition index. DI and PI were used to assess NOR; this index accounts for differences in exploration time. DI and PI are calculated as the time spent exploring (total exploration of at least 30 s, sniffing, trying to move, and front paw pushing the objects were defined as exploring, but not the time spent near the objects without investigation, or passing by the objects). Data were collected using tracking software, and manual scoring was used to assess behaviors from the videos. DI was calculated as the time spent exploring the novel object minus the time spent exploring the familiar object, divided by the total exploration time. PI was calculated as the proportion of total time spent exploring new or old object. [DI = (T_novel_ − T_familiar_)/(T_novel_ + T_familiar_), PI = T_novel_ or T_familiar_/ (T_novel_ + T_familiar_)]. All DI values fall between −1 and +1, and PI values fall between 0 and 1.

**1.14 Survival Curve Analysis**

For the aforementioned groups of mice, survival status was recorded daily from the commencement of tail vein injection until the 100^th^ day. In the records, the digit “1” denoted survival, while “0” indicated death. In this study, natural death or euthanasia due to extreme frailty was classified as a death event. All mortality events were arranged chronologically, and survival curves were plotted using the Kaplan-Meier (K-M) method. The Log-rank (Mantel-Cox) test was employed to determine significant differences in survival rates among the groups, and the Hazard Ratio was calculated using the Mantel-Haenszel method.

**1.15 Gross Necropsy and Histopathological Analysis**

After dissection, various organs including the heart, liver, kidneys, spleen, brain, and lungs of FAD3T mice treated with different regimens were examined. The corresponding tissue samples from each mouse in the single-dose toxicity study were then preserved in 4% formalin for histopathological examination. Gross lesions were also inspected and recorded in all groups. The preserved tissues were embedded in CryomatrixTM, and then 5 μm thick sections were cut and stained with hematoxylin and eosin (H&E). Histopathological examination was conducted using a Nikon Ti2 light microscope. The investigator responsible for the histopathological observations was blinded to the treatment assignment of each animal.

**1.16 In vitro BBB construction and TEER measurement**

To establish the co-culture model, bEnd.3 cells and MBVP were seeded on the top of the transwell, while C8-D1A cells were seeded on the bottom side of the dialysis chamber. The transwell was inverted so that the bottom side faced upwards for the seeding of astrocytes. The cells were permitted to attach to the lower surface of the transwell for nearly 48 hours (Figure S1A). The entire process was conducted in a sterile environment to prevent contamination during cell culture. Ultimately, the co-culture model of the three cell lines exhibiting AD-like pathology was established by incorporating HT22 cells into the wells of the culture plate. Subsequently, the transmembrane resistance values (TEER) of the cells were measured using a transendothelial electrical resistance measurement instrument. A TEER value exceeding 200 Ω⋅cm² indicates that the model has been successfully established and that subsequent permeability assays can be conducted.

**1.17 In vivo tracking**

Mice were randomly divided into 2 groups and injected with DiR-labeled free siRNA and DiR-labeled siRNA@Exo, respectively. After a single administration of the labeled siRNA or exosomes, the animals were anesthetized with 1.25% isoflurane. Fluorescence images at different time points (1, 3 and 6 hours) were acquired using the IVIS Lumina XR system at a laser power of 70–80 V. A filter (excitation at 748 nm, emission at 780 nm) was applied to capture the fluorescent images. After 24 hours, mice were sacrificed and major organs harvested to analyze drug metabolism rates. Fluorescence images of the organs were acquired using the IVIS Lumina XR system and quantitatively analyzed using Living Image 4.4 software.

**1.18 Immunofluorescence Staining of Tissue Sections**

Brain tissues were fixed with 4% paraformaldehyde for 24 hours, then dehydrated in a sucrose solution, embedded, and sectioned into 8 μm-thick frozen slices. The sections were washed three times with PBS, blocked with normal goat serum for 1 hour, and then incubated overnight at 4°C with primary antibodies including anti-NeuN (1:200, Proteintech, catalog no. 26975-1-AP), anti-RIPK3 (1:200, Abcam, ab195117), anti-MLKL (1:1000, Cell Signaling Technology, 37333T), or Phospho-Tau (Ser202, Thr205) Monoclonal antibody (AT8) (1:200, Invitrogen, MN1020). Sections were then washed three times with PBS and incubated for 1 hour at room temperature with Alexa Fluor® 594-conjugated goat anti-mouse IgG (1:200, Abcam) or Alexa Fluor® 594-conjugated goat anti-rabbit IgG (1:200, Abcam). Subsequently, the sections were stained with Hoechst33342 (1.5 μg/ml) for 10 minutes. Fluorescence images were acquired using a fluorescence microscope (Nikon Ti2), and fluorescence intensity was analyzed using ImageJ software.

**1.19 Construction and Culture of Cerebral Organoids**

The construction method for cerebral organoids is based on the modification of the protocol published by Madeline A. Lancaster's team in Nature Protocols in 2014^5^. Briefly, cultured H1, or H9 cells are dissociated into single cells and resuspended in mTeSR1 medium, then seeded into U-shaped low-adhesion 96-well plates at a density of 9000 cells per well, with Y27632 supplemented to a final concentration of 5 μM on the first day. To maintain the effect of low FGF2 concentration, SB-431542 (10 μM) and BML-275 (5 μM) are supplemented and maintained until the neural induction stage, with the culture medium being changed every other day to efficiently and uniformly initiate neural ectoderm differentiation. On the 6th day, EBs are transferred to 24-well plates using wide-mouth pipettes and cultured with Neural Induction Medium for 3 days to form neural epithelial structures, with the culture medium being changed every other day. The composition of Neural Induction Medium includes Neurobasal medium, 1:100 N2 supplement, GlutaMAX supplement, MEM non-essential amino acid solution, and 1μg/ml Heparin. On the 10th day, the neural epithelium is embedded in matrix gel and placed at 37°C for 30 minutes to solidify. After solidification, it is cultured statically with expansion medium for 3-4 days. The base composition of this medium is a 1:1 mixture of DMEM/F12 and Neurobasal medium, supplemented with 1:50 B27 supplement (without vitamin A), 1:200 N2 supplement, MEM non-essential amino acid solution, 1:100 GlutaMAX supplement, and 1:4000 Insulin. After the 14th day, it is switched to a mature medium and cultured on a shaker, with the culture medium being changed every 3-4 days. This medium is basically the same as the expansion medium mentioned above, except that the B27 supplement is replaced with B27 containing vitamin A, and add 50 mg/ml of sodium bicarbonate and 200μM of L (+)-ascorbic acid.

**1.20 Generation and Treatment of AD Organoids**

Cortical organoids were harvested between day 85 and day 90 after 12 days of serum-free treatment (control group) or 10% normal human serum treatment (AD group) for experimental analysis^6^. To validate the efficacy of the exosome complex, after 5 days of 10% serum treatment, the culture system was treated with exosome complexes loaded with si-*Ripk3* or si-*NC* for 10 days (10% Serum+si-*Ripk3*@Exo^RVG^ group, 10% Serum +Exo^RVG^@si-*NC* group), and harvested between the 85th day and the 90th day for experimental analysis.

**1.21 Statistical Analysis**

Data are presented as the mean ± standard deviation (S.D.). The normality of data distribution was assessed using histograms. For datasets that were not normally distributed, statistical significance was determined using the non-parametric Kruskal–Wallis H-test, followed by post-hoc analysis with the Mann–Whitney U-test. For datasets that satisfied the assumption of normality, a one-way analysis of variance (ANOVA) was performed. If ANOVA results show a significant main effect, Tukey 's multiple comparison test is used. Behavioral data, involving repeated measurements over time, were analyzed using a two-way repeated-measures ANOVA, with ‘treatment’ and ‘time’ (day) and their interaction as fixed factors. The specific P-values indicating statistical significance are reported in the corresponding figure legends. All statistical analyses were performed using GraphPad Prism software (version 6.0).

The supplementary data for statistical analysis are provided in an Excel file (filename: Supplementary Data for statistical analysis).

**1.22 Bulk RNA Sequencing and Bioinformatic Analysis**

To minimize variability due to sampling and processing, each biological replicate consisted of at least three mice, and all replicates for experimental conditions were processed in parallel for RNA extraction, library preparation, and sequencing. At the desired experimental endpoints, mice were anesthetized and euthanized, followed by dissection of the cerebral cortex or hippocampus. Total RNA was isolated and purified using the Total RNA Extraction Kit 2.0 Plus (Zymo Research) according to the manufacturer’s protocol. RNA concentration and integrity were assessed using a Nanodrop spectrophotometer (Agilent).

The RNA libraries were sequenced on the illumina NovaseqTM 6000 platform by OE Biotech, Inc., Shanghai, China. Bioinformatic analysis was performed using the OECloud tools at https://cloud.oebiotech.com/task/. The volcano map (or other graphics) was drawn based on the R (https://www.r-project.org/) on the OECloud platform ( <https://cloud.oebiotech.com/task/>).

*Raw Data Quality Control and Sequence Alignment* The raw sequencing data underwent comprehensive quality control preprocessing using the fastp software. This process included the removal of adapter and primer sequences, filtering of reads containing N bases, discarding low-quality reads (Qphred < 20), and performing sliding window quality trimming at both ends of the sequences to obtain high-quality Clean Reads. Subsequently, the Clean Reads were aligned to the mouse reference genome (GRCm38/mm10) using hisat2 (Hierarchical Indexing for Spliced Alignment of Transcripts 2) to generate BAM files required for transcript assembly and quantification. Based on the alignment results and the Gene Transfer Format (GTF) annotation file, the htseq-count software was used to count the number of read fragments uniquely mapped to the exonic regions of each gene, thereby constructing a raw gene expression count matrix.

*Differential Gene Expression Analysis* The identification of differentially expressed genes (DEGs) was performed using the DESeq2 software package. This algorithm first normalizes the raw count data based on a negative binomial model to correct for library size and gene length biases. It employs a shrinkage estimation method to improve the accuracy of log_2_ fold change (log_2_FC) calculations. The statistical significance of differential expression was assessed using the Wald test, and p-values were adjusted for multiple hypothesis testing using the Benjamini-Hochberg (BH) method to control the false discovery rate (FDR). The screening criteria for DEGs were set as FDR < 0.05 and |log_2_FC| > 1. Based on the identified DEGs, a volcano plot was generated using the OECloud platform to visualize their overall distribution. Concurrently, unsupervised hierarchical clustering analysis was performed based on the expression levels of DEGs, and a heatmap was generated to assess the transcriptional similarity among samples.

*Functional and Pathway Enrichment Analysis* To systematically elucidate the biological functions of the DEGs, we performed Gene Ontology (GO) and Kyoto Encyclopedia of Genes and Genomes (KEGG) enrichment analyses. The GO analysis was conducted across three ontological domains—Biological Process, Cellular Component, and Molecular Function. The significance of DEG enrichment in each GO term was calculated using a hypergeometric test (p-value). The KEGG pathway enrichment analysis aimed to identify signaling pathways in which the DEGs were significantly enriched, with enrichment significance also assessed by p-value. All enrichment analysis results were visualized using the OECloud platform, generating bar charts or bubble plots to intuitively display key functional terms and pathways.

The raw sequence data have been submitted to the NCBI Gene Expression Omnibus (GEO) datasets with accession number < GSE315110 >.

**2. Supplementary figures and Table S1-S3**

**Supplementary figure 1, Validation of a blood-brain barrier model and demonstration of efficient, biocompatible neuronal uptake of Exo^RVG^ exosomes. A** Schematic timeline illustrating the experimental protocol, including cell seeding phases, co-culture establishment, and the analysis window when permeability assessments were conducted. **B** Trans-epithelial electrical resistance (TEER) measurements over the 8-day culture period. **C** Schematic representation of tracer transport assay using PKH26-labeled exosomes. **D** Quantitative Results of Tracer Distribution. **E** Compared to 293T cells, HT22 cells are capable of effectively uptakeing Exo**^RVG^**. Nuclei: Hoechst 33342 (blue), cytoplasm: GFP (green), Exo^RVG^ (PKH26, red). In **(a-j)**, scale bar = 200 µm. **(i-j)** show magnified views of the boxed areas in **(g-h),** respectively. **F** The effect of Exo and Exo**^RVG^** on the viability of HT22 neuronal cells. HT22 neuronal cells were treated with cell culture medium (control), control Exo, or Exo^RVG^ for 24 hours, and then the viability was measured according to the CCK-8 assay protocol. Data are presented as mean ± SD (*n*=5). Statistical analysis was performed by one-way ANOVA followed by Tukey’s post hoc test.

**Supplementary figure 2, Si-*Ripk3*@Exo^RVG^ inhibits the increase of pMLKL expression in HT22 cells induced by OA.**

**Supplementary figure 3, Si-*Ripk3*@Exo^RVG^ inhibits the increase of pRIPK3 expression in HT22 cells induced by OA.**

**Supplementary figure 4, General behavioral performance of mice in nesting and OFT. A** Representative images of the nesting experiments for each group. **B**-**D** The results of the OFT experiment. B Trajectory graphs of mice in the OFT. **C** The total distance moved by each group of mice in the OFT. **D** The graph depicting the number of times each group of mice stood in the OFT. All statistics were analyzed using one-way ANOVA followed by Tukey’s post hoc test.

**Supplementary figure 5, Treatment with si-*Ripk3*@Exo^RVG^ significantly inhibited the activation of microglia and astrocytes in the brain.** **A** Immunofluorescence staining shows that si-*Ripk3*@Exo^RVG^ treatment significantly inhibits the activation of astrocytes (GFAP, red) in the brain of AD model mice. Blue indicates cell nuclei (Hoechst 33342). Scale bar = 50 μm. **B** Quantitative analysis indicates that si-*Ripk3*@Exo^RVG^ treatment significantly reduces the number of branch points in astrocytes. **C** Quantitative analysis indicates that si-*Ripk3*@Exo^RVG^ treatment significantly decreases the soma area of astrocytes. **D** Three-dimensional reconstruction images of microglia (IBA1) visually demonstrate the ameliorative effect of si-*Ripk3*@Exo^RVG^ on their morphology.

**Supplementary figure 6, Si-*Ripk3*@Exo^RVG^ effectively inhibits the neurotoxicity of pTau.** **A** Representative images of AT8 staining (red) for phospho-tau in the hippocampus and cortex of WT-Vehicle, FAD-Vehicle, and FAD-si-*Ripk3*@Exo^RVG^ mice; nuclei counterstained with Hoechst 33342 (blue). Scale bar: 100 μm. **B** Quantification of AT8-positive area in the ROI, showing reduced tau hyperphosphorylation in si-*Ripk3*@Exo^RVG^-treated mice vs. FAD-Vehicle controls. **C** Western blot analysis of total Tau and phospho-Tau (S202/T205) in brain lysates. **D** Quantification shows reduced pTau/Tau ratio in Si-Ripk3@ExoRVG-treated mice. Statistical analysis was performed using one-way ANOVA followed by Tukey’s post hoc test.

**Supplementary figure 7, Transcriptional regulation by si-*Ripk3*@Exo^RVG^ enhances synaptic plasticity and cortical neuron survival in mice. A** The volcano plot shows the DEGs between the si-*Ripk3*@Exo^RVG^ treatment group and the control group. Upregulated genes are indicated in red, and downregulated genes are indicated in blue. The screening criteria were set as p-value < 0.05 and |log_2_FC| > 1. **B** Hierarchical clustering heatmap of DEGs. **C** Bar chart of Gene Ontology (GO) enrichment analysis, showing the top 25 significantly enriched Biological Processes for the differentially expressed genes. **D** The bubble plot shows the KEGG pathway enrichment analysis of the top 15 significantly downregulated signaling pathways following si-*Ripk3*@Exo^RVG^ treatment. These pathways are predominantly enriched in inflammation- and neurotoxicity-related processes. In the plot, the bubble size represents the number of enriched differentially expressed genes, and the color indicates the significance of enrichment (-log_10_ p-value). **E** The bubble plot of KEGG pathway enrichment analysis shows the top 13 significantly upregulated signaling pathways. These pathways are predominantly enriched in processes related to synaptic function and neuroprotection.

**Supplementary figure 8, Construction and characterization of human cortical organoids. A** Schematic representation of the process for constructing and treating cortical organoids. **B** Representative image of co-staining for TUJ1 and SOX2 immunofluorescence. **C** Representative image of co-staining for Ki67 and PAX6 immunofluorescence. **D** Representative image of co-staining for ZO-1 and PAX6 immunofluorescence. The white dashed circle delineates the ventricular zone (VZ).

**Supplementary figure 9, Characterizing the maturity of human cortical organoids by immunofluorescence. A** Representative image of co-staining for TBR1 and PAX6 immunofluorescence. The white dashed circle delineates the ventricular zone (VZ). VL: ventricular lumen; CP: cortical plate. **B** Representative image of co-staining for CUX1 and SATB2 immunofluorescence. **C** Representative image of MAP2 immunofluorescence. **D** Representative image of GFAP immunofluorescence. **E** A schematic diagram of the cortical structure of organoids. The diagram summarizes the hierarchical structure of organoids from the ventricular zone (VZ) to the cortical plate (CP), including PAX6⁺/SOX2⁺/Ki67⁺ neural stem cells, TBR1⁺ deep-layer neurons, CUX1⁺/SATB2⁺ upper-layer neurons, and GFAP⁺ astrocytes, as well as the neural network structure marked by MAP2, intuitively demonstrating the similarity between organoids and in vivo cortical development.

**Supplementary figure 10, Construction of RVG engineered exosomes from human system. A** The expression level of LAMP2 protein in HEK293T-RVG cells was detected by Western blot. (left) representative immunoblotting, (right) Quantitative statistical analysis (*n*=6). Comparisons between two groups were performed using a *t*-test. **B** The particle size detection results of exosomes from human system. **C** Average particle size statistics of exosomes before and after electroporation loading siRNA (*n*=3). Comparisons between two groups were performed using a *t*-test.

**Supplementary figure 11, qPCR screening of siRNA (human) sequences.** As shown in the figure, si-*Ripk3*-1352 showed a silencing rate of about 70 %. Statistical analysis was performed using one-way ANOVA followed by Tukey’s post hoc test..

**Supplementary figure 12, Development and Characterization of Exo^RVG^.** **A** Schematic diagram showing the steps involved in the production of engineered Exo^RVG^. **B** Key elements of the plasmid used in the development of engineered exosomes. **C** Protein levels of Flag in control and RVG-Lamp2-Flag overexpressing HT22 hippocampal neuron cells. **D** Representative particle size distribution of Exo^RVG^. **E** Comparison of size between control Exo and Exo^RVG^, *n*=3. **F-G** Representative transmission electron microscopy (TEM) images, with (**G)** showing a magnified view of a specific area. **H** Exosome marker protein levels (CD63, TSG101, Calnexin) determined by Western Blot. **I-J** Display of exosome zeta potential and electrophoretic mobility. Data are presented as mean ± standard deviation (SD), *n*=3. Statistical analysis was performed by Student's *t*-test to compare Exo vs. Exo^RVG^. Data points represent independently prepared exosomes.

**Supplementary figure 13,** **In vivo imaging demonstrates the efficient BBB penetration capability of siRNA@Exo. A** Schematic of the in vivo imaging experimental workflow. **B** Representative in vivo images at 1, 3 and 6 h post-injection. **C** Quantification of total radiant efficiency within the ROI. Brain quantification was performed using ROIs (red circles in panel B) to specifically capture the brain accumulation signal. Data were obtained and analyzed using Living Image 4.4 software, *n*=3. **D** Ex vivo imaging of organs and quantification of total radiant efficiency at 24 h post-injection. The left panel of Figure D shows ex vivo organ imaging at 24 h, and the right panel shows the results of quantitative analysis, *n*=3. Data were analyzed using a two-tailed *t*-test.

**Supplementary figure 14, Cytotoxicity and in vivo biocompatibility assessment of the si-*Ripk3*@Exo^RVG^ nanomedicine. A** Representative data of hematoxylin and eosin (HE) staining of major organs from APP/PS1/TAU mice treated with si-*Ripk3*@Exo^RVG^ or vehicle and control wild-type (WT) mice in 15 injection treatment experiments. **B** and **C** Blood biochemical examination. Assessment of alanine transaminase (ALT), aspartate transaminase (AST), plasma urea (BUN), creatinine (CR), and uric acid (UA) levels in plasma after nanomedicine treatment with a single dose. *n*=7, mean with SD. **D** and **E** The expression levels of liver and kidney mRNA. In the liver (**D**) and kidney (**E**), the levels of key pro-inflammatory cytokines (such as IL-1β, IL-6, and TNF-α) were assessed following a single administration of vehicle or si-*Ripk3*@Exo^RVG^ treatment. *n*=3, mean with SD. Data points represent independent replicates, and statistical analysis was performed using an unpaired *t*-test. Note: Since WT mice lack the disease baseline necessary for assessing treatment-induced toxicity, they were excluded from this specific assay to minimize animal use in accordance with the 3R principles.

**Supplementary figure 15, Si-*Ripk3*@Exo^RVG^ enhances synaptic plasticity and promotes hippocampal neuron survival via transcriptional regulation. A** Schematic diagram of the workflow for tissue collection and transcriptome sequencing analysis. **B** The volcano plot shows the differentially expressed genes (DEGs) between the si-*Ripk3*@Exo^RVG^ treatment group and the control group. Upregulated genes are indicated in red, and downregulated genes are indicated in blue. The screening criteria were set as p-value < 0.05 and |log_2_FC| > 1. **C** Hierarchical clustering heatmap of DEGs. **D** Bar chart of Gene Ontology (GO) enrichment analysis, showing the top 21 significantly enriched Biological Processes and the top 7 Cellular Components for the differentially expressed genes. **E** The bubble plot shows the KEGG pathway enrichment analysis of the top 15 significantly downregulated signaling pathways following si-*Ripk3*@Exo^RVG^ treatment. These pathways are predominantly enriched in inflammation- and neurotoxicity-related processes. In the plot, the bubble size represents the number of enriched differentially expressed genes, and the color indicates the significance of enrichment (-log10 p-value). **F** The bubble plot of KEGG pathway enrichment analysis shows the top 15 significantly upregulated signaling pathways. These pathways are predominantly enriched in processes related to synaptic function and neuroprotection.

**Supplementary figure 16, Si-*Ripk3*@Exo^RVG^ protects neurons by activating PKG and inhibiting p38. A** Si-*Ripk3*@Exo^RVG^ inhibits p38 MAPK activation. Western blot analysis was performed to assess the phosphorylation status of p38 MAPK (p-p38(Y182)) in HT22 cells treated with oxaloacetic acid (OA), Exo^RVG^, or si-*Ripk3*@Exo^RVG^. The graph on the right shows the quantification of the p-p38/p38 ratio, normalized to the control group. Data are presented as mean ± SD (*n*=10). **B** Si-*Ripk3*@Exo^RVG^ inhibits ERK1/2 MAPK activation. Western blot analysis was used to evaluate the phosphorylation of ERK1/2 (p-ERK1/2) in HT22 cells under the same treatment conditions. The graph on the right quantifies the p-ERK1/2/ERK1/2 ratio. Treatment with si-*Ripk3*@Exo^RVG^ did not alter ERK1/2 phosphorylation. Data are presented as mean ± SD (*n*=9). **C** Si-*Ripk3*@Exo^RVG^ inhibits VASP phosphorylation. The phosphorylation of VASP (p-VASP(S239)), a downstream effector of the PKG pathway and a marker of actin cytoskeleton remodeling, was analyzed via Western blot. The graph on the right shows the quantification of the p-VASP/VASP ratio. Si-*Ripk3*@Exo^RVG^ treatment led to a significant reduction in VASP phosphorylation, suggesting a restoration of normal cytoskeletal dynamics. Data are presented as mean ± SD (*n* = 5). Data points represent an independent replicate, and statistical analysis was performed using one-way ANOVA followed by Tukey’s post hoc test. **D** Si-*Ripk3*@Exo^RVG^ confers synergistic neuroprotection by suppressing neuronal necroptosis, activating cGMP-PKG signaling, and inhibiting p38 MAPK in an AD mouse model.

**Supplementary figure 17, Exo@si-*Ripk3* alleviates serum-induced necroptosis and Alzheimer’s disease-like pathology in cortical organoids. A** Representative image of co-staining for TAU and AT8 immunofluorescence. AT8 stains for phosphorylated Tau (Ser202, Thr205). **B** Fold change in AT8 fluorescence intensity is expressed as the AT8/Tau ratio and normalized to the control. Data are presented as mean ± SD (*n*=3). **C** Representative original Western blot bands and quantitative analysis of phosphorylated Tau. Data are presented as mean ± SD (*n*=9). **D** Representative image of phosphorylated Tau (Ser396) immunofluorescence staining. **E** Representative original Western blot bands of phosphorylated MLKL(Ser358) and MLKL. **F** The bar graph shows the quantitative analysis results of the pMLKL/MLKL ratio. Data are presented as mean ± SD (*n*=4). Statistical analysis was performed using one-way ANOVA followed by Tukey’s post hoc test. Data points in panel B represent individual organoids, and in panels C and F, Western blot band quantifications from independent replicates.

**Table S1.** Specifications of antibodies used in the present study

| **Antibody name** | **Source / Company** | | **Catalogue number** | | **Assays and dilution** | |
| --- | --- | --- | --- | --- | --- | --- |
| Phospho-Tau (Ser202, Thr205) Monoclonal antibody (AT8) | Invitrogen, Waltham, MA, USA | | MN1020 | | Western blotting  (WB) 1:2000 | |
| Phospho-Tau (Ser202, Thr205) Monoclonal antibody (AT8) | Invitrogen, Waltham, MA, USA | | MN1020 | | IHC (F), 1:200 | |
| Anti-Tau (phospho S396) Antibody | Abcam, Cambridge, United Kingdom | | ab109390 | | WB, 1:20000 | |
| Anti-NeuN antibody | Proteintech, Chicago, USA | | 26975-1-AP | | IHC (F), 1:200 | |
| Aniti-Tau antibody | Abmart, Shanghai, China | | T55846S | | WB, 1:1000 | |
| Anti-RIP3 (phospho S232) antibody | Abcam, Cambridge, United Kingdom | | ab195117 | | IHC (F)/ICC, 1:200 | |
| Anti-MLKL (phospho S358) antibody | Abcam, Cambridge, United Kingdom | | ab187091 | | IHC (F)/ICC, 1:100 | |
| Anti-MLKL (phospho S358) antibody | Abcam, Cambridge, United Kingdom | | ab187091 | | WB, 1:1000 | |
| Anti-PSD95 antibody | Abcam, Cambridge, United Kingdom | | ab18258 | | WB, 1:1000 | |
| Anti-ACTB/β-actin antibody | Proteintech, Chicago, USA | | 66009-1-Ig | | WB, 1:20000 | |
| Anti-β-Tubulin antibody | Proteintech, Chicago, USA | | 66240-1-Ig | | WB, 1:20000 | |
| Anti-β-Tubulin antibody | Proteintech, Chicago, USA | | 66240-1-Ig | | ICC, 1:500 | |
| Anti-Flag-Tag antibody | Proteintech, Chicago, USA | | 66008-4-Ig | | WB, 1:5000 | |
| Anti-TSG101 antibody | Proteintech, Chicago, USA | | 28283-1-AP | | WB, 1:1000 | |
| Anti-CD63 antibody | Proteintech, Chicago, USA | | 25682-1-AP | | WB, 1:1000 | |
| Anti-Calnexin antibody | Proteintech, Chicago, USA | | 10427-2-AP | | WB, 1:5000 | |
| Anti-MLKL antibody | Proteintech, Chicago, USA | | 21066-1-AP | | WB, 1:000 | |
| Anti-RIPK1 antibody | Proteintech, Chicago, USA | | 17519-1-AP | | WB, 1:000 | |
| Anti-Pro-Caspase-8 antibody | Abcam, Cambridge, United Kingdom | | ab108333 | | WB, 1:1000 | |
| Anti-p38 MAPK antibody | Abclonal, China | | A5049 | | WB, 1:1000 | |
| Anti-p38 MAPK (phospho Y182) antibody | Abclonal, China | | AP1372 | | WB, 1:1000 | |
| Anti-ERK1/2 antibody | Abclonal, China | | A4782 | | WB, 1:1000 | |
| Anti-ERK1/2 (phosphor T202/Y204+T185/Y187) antibody | Abclonal, China | | AP0472 | | WB, 1:1000 | |
| Anti-VASP antibody | Abclonal, China | | A14217 | | WB, 1:1000 | |
| Anti-VASP (phosphor S239) antibody | Affinity，China | | AF3338 | | WB, 1:1000 | |
| Anti-ZO-1 antibody | Proteintech | | 21773-1-AP | | IHC (F), 1:200 | |
| Anti-Tau (phospho S396) antibody | Abcam | | ab109390 | | IHC (F), 1:200 | |
| Anti-TUJ1 antibody | Abcam | ab68193 | | IHC (F), 1:200 | |  |
| Anti-SATB2 antibody | Abmart | MU140452S | | IHC (F), 1:200 | |  |
| Anti-SOX2 antibody | Abcam | | ab79351 | | IHC (F), 1:200 | |
| Anti-MAP2 antibody | Abclonal | | A22206 | | IHC (F), 1:200 | |
| Anti-PAX6 antibody | GeneTex | | GT9412 | | IHC (F), 1:200 | |
| Anti-Ki67 Antibody | Abmart | | TW0001S | | IHC (F), 1:200 | |
| Anti-TBR1 antibody | Abcam | ab183032 | | IHC (F), 1:200 | |  |
| Anti-CUX1 antibody | Abmart | PA1303S | | IHC (F), 1:200 | |  |
| Anti-GRIA1 antibody | Proteintech | 67642-Ig | | IHC (F), 1:200 | |  |
| Anti-GFAP antibody | Proteintech | 16825-1-AP | | IHC (F), 1:200 | |  |

**Table S2.** qPCR and PCR primer sequences used in the study

| Target gene | Forward/ Reverse | Primer sequences（5'-3'） |
| --- | --- | --- |
| Mouse *Tnfα* | Forward | 5'- CCCTCACACTCACAAACCAC-3' |
|  | Reverse | 5'- ACAAGGTACAACCCATCGGC-3' |
| Mouse *Il-6* | Forward | 5'- TGGTCTTCTGGAGTACCATAGC-3' |
|  | Reverse | 5'- TGTGACTCCAGCTTATCTCTTGG-3' |
| Mouse *Il-1β* | Forward | 5'- TGCCACCTTTTGACAGTGATG-3' |
|  | Reverse | 5'- TGATGTGCTGCTGCGAGATT-3' |
| Mouse *Ripk3* | Forward | 5'CTCCGTGCCTTGACCTACTG-3' |
|  | Reverse | 5'CTCACCAGAGGAACCGCATA-3' |
| Human *Ripk3* (qPCR) | Forward | 5'-AAATGCCCGAGCCTTACC-3' |
|  | Reverse | 5'-GCCTTCTTGCGAACCTACT-3' |
| Human *Lamp2* (PCR) | Forward | 5'-CCGCTCGAGATGGTGTGCTTCCGCCT-3' |
|  | Reverse | 5'-ATAAGAATGCGGCCGCTTACACAGACTGATAACCAGTACGA-3' |

**Table S3.** siRNA sequences used in this study

| siRNA | Sense/Anti-sense | Oligo序列（5'-3'） |
| --- | --- | --- |
| si-*Ripk3*-783 (mouse) | Sense | AGCUGUUAUUUGAUGUCAA/dT//dT/ |
|  | Anti-sense | UUGACAUCAAAUAACAGCU/dT//dT |
| si-*Ripk3-*883(mouse) | Sense | GGUAGACAAGACUUCACUA/dT//dT |
|  | Anti-sense | UAGUGAAGUCUUGUCUACC/dT//dT |
| si-*Ripk3*-1103 (mouse) | Sense | GCUGCUGUCUCCGAGGUAA/dT//T/ |
|  | Anti-sense | UUACCUCGGAGACAGCAGC/dT//dT/ |
| si-*Ripk3*-728 (human) | Sense | GAACUGUUUGUUAACGUAA/dT//dT/ |
|  | Anti-sense | UUACGUUAACAAACAGUUC/dT//dT/ |
| si-*Ripk3*-1352 (human) | Sense | CAGACUCCAGAGACCUCAA/dT//dT/ |
|  | Anti-sense | UUGAGGUCUCUGGAGUCUG/dT//dT/ |
| si-*Ripk3*-1552 (human) | Sense | CAACUACUUGACUAUGCAA/dT//dT/ |
|  | Anti-sense | UUGCAUAGUCAAGUAGUUG/dT//dT/ |
| si-*Ripk3*-*NC* | Sense | UUCUCCGAACGUGUCACGU/dT//dT/ |
|  | Anti-sense | ACGUGACACGUUCGGAGAA/dT//dT/ |

**Reference**

1	Li, Y. *et al.* An anti-FAP-scFv-functionalized exosome-carrying hydrogel delivers SKI mRNA to fibrotic nucleus pulposus cells to alleviate intervertebral disc degeneration by regulating FOXO3. *Theranostics* **15**, 3877-3899 (2025). <https://doi.org:10.7150/thno.107776>

2	He, S. *et al.* Biomimetic gene delivery system coupled with extracellular vesicle-encapsulated AAV for improving diabetic wound through promoting vascularization and remodeling of inflammatory microenvironment. *J Nanobiotechnology* **23**, 242 (2025). <https://doi.org:10.1186/s12951-025-03261-w>

3	Han, J. *et al.* Engineered exosomes with a photoinducible protein delivery system enable CRISPR-Cas-based epigenome editing in Alzheimer's disease. *Sci Transl Med* **16**, eadi4830 (2024). <https://doi.org:10.1126/scitranslmed.adi4830>

4	Wesson, D. W. & Wilson, D. A. Age and gene overexpression interact to abolish nesting behavior in Tg2576 amyloid precursor protein (APP) mice. *Behav Brain Res* **216**, 408-413 (2011). <https://doi.org:10.1016/j.bbr.2010.08.033>

5	Lancaster, M. A. & Knoblich, J. A. Generation of cerebral organoids from human pluripotent stem cells. *Nat Protoc* **9**, 2329-2340 (2014). <https://doi.org:10.1038/nprot.2014.158>

6	Chen, X. *et al.* Modeling Sporadic Alzheimer's Disease in Human Brain Organoids under Serum Exposure. *Adv Sci (Weinh)* **8**, e2101462 (2021). <https://doi.org:10.1002/advs.202101462>
